# One-pot stereoselective synthesis of α,β-differentiated diamino esters via the sequence of aminochlorination, aziridination and intermolecular S_N_2 reaction

**DOI:** 10.3762/bjoc.10.189

**Published:** 2014-08-07

**Authors:** Yiwen Xiong, Ping Qian, Chenhui Cao, Haibo Mei, Jianlin Han, Guigen Li, Yi Pan

**Affiliations:** 1 School of Chemsitry and Chemical Engineering, State of Key Laboratory of Coordination, Nanjing University, Nanjing, 210093, China; 2 Institute for Chemistry & BioMedical Sciences, Nanjing University, Nanjing, 210093, China; 3 High-Tech Research Institute of Nanjing University, Changzhou, 213164, China; 4 Department of Chemsitry and Biochemistry, Texas Tech Unviersity, Lubbock, Texas, 79409-1061, USA

**Keywords:** aminohalogenation, diamination, α,β-diamino ester, one-pot, stereoselectivity

## Abstract

We report here an efficient one-pot method for the synthesis of α,β-differentiated diamino esters directly from cinnamate esters using *N*,*N*-dichloro-*p*-toluenesulfonamide and benzylamine as nitrogen sources. The key transformations include a Cu-catalyzed aminohalogenation and aziridination, followed by an intermolecular S_N_2 nucleophilic ring opening by benzylamine. The reactions feature a wide scope of substrates and proceed with excellent stereo- and regioselectivity (*anti*:*syn* >99:1) .

## Introduction

α,β-Diamino acid derivatives are one of the most important classes of nitrogen-containing bioactive compounds [1–2]. Their chemistry has attracted a lot of attention [3–5], leading to discovery and development of numerous useful compounds in the fields of biology, medicine, therapy and food [6–9]. They also belong to useful organic synthetic intermediates as they can be easily converted into the corresponding β,γ-amino alcohols and vicinal diamines. α,β-Diamino acid derivatives have been served as organocatalysts, chiral ligands, chiral auxiliaries for asymmertric synthesis [10–12], as well as synthetic fragments for peptides and natural products [13].

Mannich-type addition reactions of α-amino acid derivatives with imino compounds, or their precursors, is one of the most straightforward synthetic approaches to α,β-diamino acid compounds, in particular in asymmetric mode [14–22]. Direct catalytic oxidative diaminations of functionalized alkenes also present an access for the generation of α,β-diamino esters, which usually employ palladium or osmium as catalysts [23–25]. The electrophilic diamination reaction is an alternative methodology [26–28], which makes use of α,β-unsaturated esters as starting materials to form imidazoline diamine derivatives. However, these methods suffer from the shortcomings, such as need of special starting materials, use of expensive metal catalysts or strict anhydrous and anaerobic conditions.

**Scheme 1:**
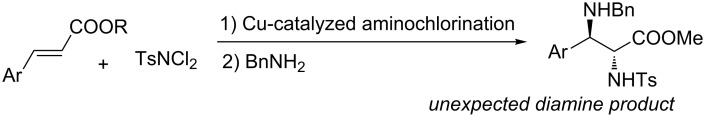
An anomalous outcome with benzylamine as organic base.

The aminohalogenation reaction has been well studied in the past decade [29–32], and the corresponding vicinal haloamine product can be easily converted into aziridines [33–34] and α,β-dehydroamino acid derivatives [35] in the presence of an organic amine. Recently, we found that treating haloamine with benzylamine resulted in an unexpected α,β-diamino product, instead of the aziridine or the α,β-dehydroamino product. Herein, we report an anomalous outcome in the one-pot reaction, which provides a highly efficient method for the synthesis of α,β-differentiated diamino esters directly from readily available starting materials, α,β-unsaturated ester, *N*,*N*-dichlorotoluenesulfonamide (TsNCl_2_) and benzylamine. Furthermore, the reaction could be conducted in a one-pot model, under operationally convenient conditions [36–39] through Cu-catalyzed aminohalogenation, aziridination and intermolecular S_N_2 nucleophilic ring opening without isolation of haloamine intermediate (Scheme 1).

## Results and Discussion

**Scheme 2:**
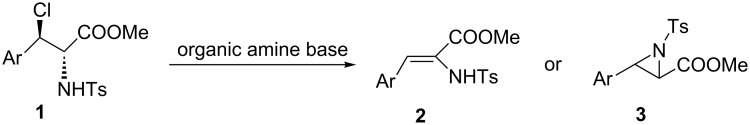
Transformation of vicinal haloamines by the use of organic amines.

According to the previous reports on the derivatization of aminohalogenation reactions, the vicinal haloamines usually underwent elimination or aziridination reactions when they were treated with organic bases (Scheme 2) [33–35]. However, when benzylamine was added to haloamine **1a** in acetonitrile, the reaction could also proceed smoothly giving a sole product. Quite unexpectedly, the ^1^H NMR data showed the presence of a benzyl group. This result clearly indicated that the benzylamine substituted product was formed.

Encouraged by this result, we then focused on the optimization of the reaction conditions with **1a** as a model substrate to fully explore this new synthetic method (Table 1). Diamine product **5a** was obtained in 83% yield when **1a** reacted with benzylamine in acetonitrile at room temperature for 0.5 h (Table 1, entry 1). Increasing the temperature to 50 °C, gave no improvement on the yield (Table 1, entry 2). A higher yield was obtained when the reaction time was prolonged to 1 h (Table 1, entry 3). Further optimization efforts showed that the base loading amount could be lowered to 2 mL without any drop in yield (Table 1, entries 4 and 5). When 0.1 mL of benzylamine was used for this transformation in the presence of 2 mL triethylamine, the yield decreased dramatically even the reaction time was prolonged to 6 h (Table 1, entries 6–8). The solvent was also proved to be crucial for this transformation (Table 1, entries 4, 9 and 10). As shown by these experiments, acetonitrile and dichloromethane were the best choices. With the aim of developing a one-pot method, we chose acetonitrile as solvent for the following experiments because the previous reports indicated acetonitrile was the best solvent for the aminohalogenation of methyl cinnamate (**4a**).

**Table 1:** Optimization of typical reaction conditions.^a^

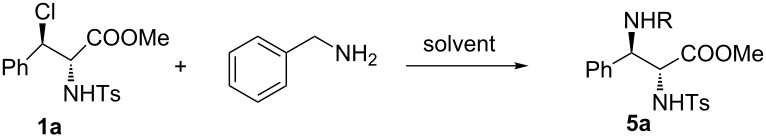					

entry	amount (mL)^b^	solvent	*T* (°C)	time (h)	yield (%)^c^

1	4	CH_3_CN	rt	0.5	83
2	4	CH_3_CN	50	0.5	75
3	4	CH_3_CN	rt	1	91
4	2	CH_3_CN	rt	1	93
5	0.5	CH_3_CN	rt	1	63
6	0.1	CH_3_CN	rt	1	28^d^
7	0.1	CH_3_CN	rt	3	59^d^
8	0.1	CH_3_CN	rt	6	60^d^
9	2	CH_2_Cl_2_	rt	1	89
10	2	CHCl_3_	rt	1	80

^a^Reaction conditions: **1a** (0.5 mmol), solvent (3 mL). ^b^Amount of benzylamine. ^c^ Isolated yields. ^d^2 mL triethylamine was added.

To prove the synthetic value of the methodology, other common primary or secondary amines, were tested in the reaction under optimized conditions (Table 2). The use of aliphatic amines, such as methylamine (Table 2, entry 2), dimethylamine (Table 2, entry 3) and ammonia solution (Table 2, entry 4), lead to the formation of the aziridine as the sole product in 88%, 83%, 91% yield, respectively. Notably, a complex mixture was obtained when 1,2-ethanediamine was used in this reaction (Table 2, entry 1).

**Table 2:** Examination of other organic bases.^a^

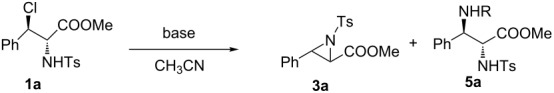					

entry	base (mL)	*T* (°C)	time (min)	product (%)^b^	

				**3a**	**5a**

1	1,2-ethanediamine (2)	rt	30	complex mixture	
2	methylamine (2)	rt	30	88	
3	dimethylamine (2)	rt	30	83	
4	ammonia solution (2)	rt	30	91	

^a^Reaction conditions: **1a** (0.5 mmol), acetonitrile (3 mL), base. ^b^Isolated yields.

After getting the optimized conditions, we then combined the aminohalogenation and the treatment of benyzlamine to develop a one-pot procedure with α,β-unsaturated esters as starting materials. On the initial reaction step the cinnamic ester underwent a copper(II) trifluoromethanesulfonate-catalyzed aminohalogenation reaction with TsNCl_2_ as nitrogen source. After being quenched by saturated sodium sulfite, the resulting mixture was stirred with benzylamine. Various α,β-unsaturated esters were studied to evaluate the yield and stereochemical outcome of these reactions (Table 3). As shown in Table 3, almost all of the tested substrates worked well under the optimized conditions giving rise to the corresponding α,β-diamino ester products, even though the aromatic ring was substituted by strong electron-withdrawing groups (fluoro, Table 3, entries 6, 10 and 12; trifluoromethyl, entry 15) or an electron-donating group (methoxy, Table 3, entry 8). In the case of ethyl ester, the reaction showed lower reactivity (Table 3, entry 2), and 70% chemical yield was obtained comparing to 79% yield from methyl ester (Table 3, entry 1). A cinnamic ester with double-substituted aromatic ring **4m** was also tolerated in this reaction along with a moderate chemical yield (53%, Table 3, entry 13). Notably, when the phenyl was replaced by 1-naphthyl **4n** (Table 3, entry 14), it was also well performing in this reaction giving rise to the target product in 64% yield. For the substrates with *ortho*-substituents (Table 3, entries 13 and 16), the yields were a little bit lower than the yields of the *meta*- and *para*-substituted substrates, which indicates that the steric hindrance affects the formation of the product. Furthermore, excellent stereoselectivity was obtained for all of the examined cinnamic ester substrates, and only the *anti*-isomers were observed.

**Table 3:** One-pot reaction for the synthesis of α,β-diamino ester.^a^

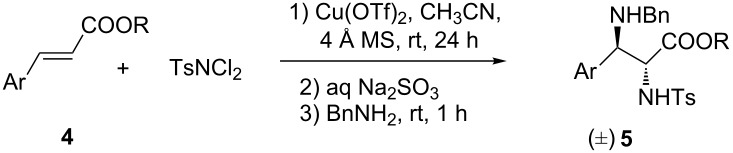					

entry	Ar	R	product	yield (%)^b^	*anti*:*syn*^ c^

1	C_6_H_5_	Me	**5a**	79	>99:1
2	C_6_H_5_	Et	**5b**	70	>99:1
3	4-CH_3_-C_6_H_4_	Me	**5c**	67	>99:1
4	4-Br-C_6_H_4_	Me	**5d**	72	>99:1
5	4-Cl-C_6_H_4_	Me	**5e**	68	>99:1
6	4-F-C_6_H_4_	Me	**5f**	78	>99:1
7	4-CF_3_O-C_6_H_4_	Me	**5g**	80	>99:1
8	3-CH_3_O-C_6_H_4_	Me	**5h**	70	>99:1
9	3-Cl-C_6_H_4_	Me	**5i**	67	>99:1
10	3-F-C_6_H_4_	Me	**5j**	75	>99:1
11	2-Cl-C_6_H_4_	Me	**5k**	63	>99:1
12	2-F-C_6_H_4_	Me	**5l**	83	>99:1
13	2,6-di-Cl-C_6_H_3_	Me	**5m**	53	>99:1
14	1-naphthyl	Me	**5n**	64	>99:1
15	3-CF_3_-C_6_H_4_	Me	**5o**	74	>99:1
16	2-Br-C_6_H_4_	Me	**5p**	58	>99:1

^a^Reaction conditions: 1) 10 mol % Cu(OTf)_2_, 0.5 mmol cinnamic ester **4**, 1.0 mmol TsNCl_2_, 250 mg 4 Å molecular sieves in 3.0 mL acetonitrile at room temperature for 24 h; 2) Quenched by 3 mL saturated Na_2_SO_3_ for 30 min; 3) Benzylamine 2.0 mL at room temperature for 1 h. ^b^Isolated yield. ^c^Determined by ^1^H NMR.

**Figure 1:**
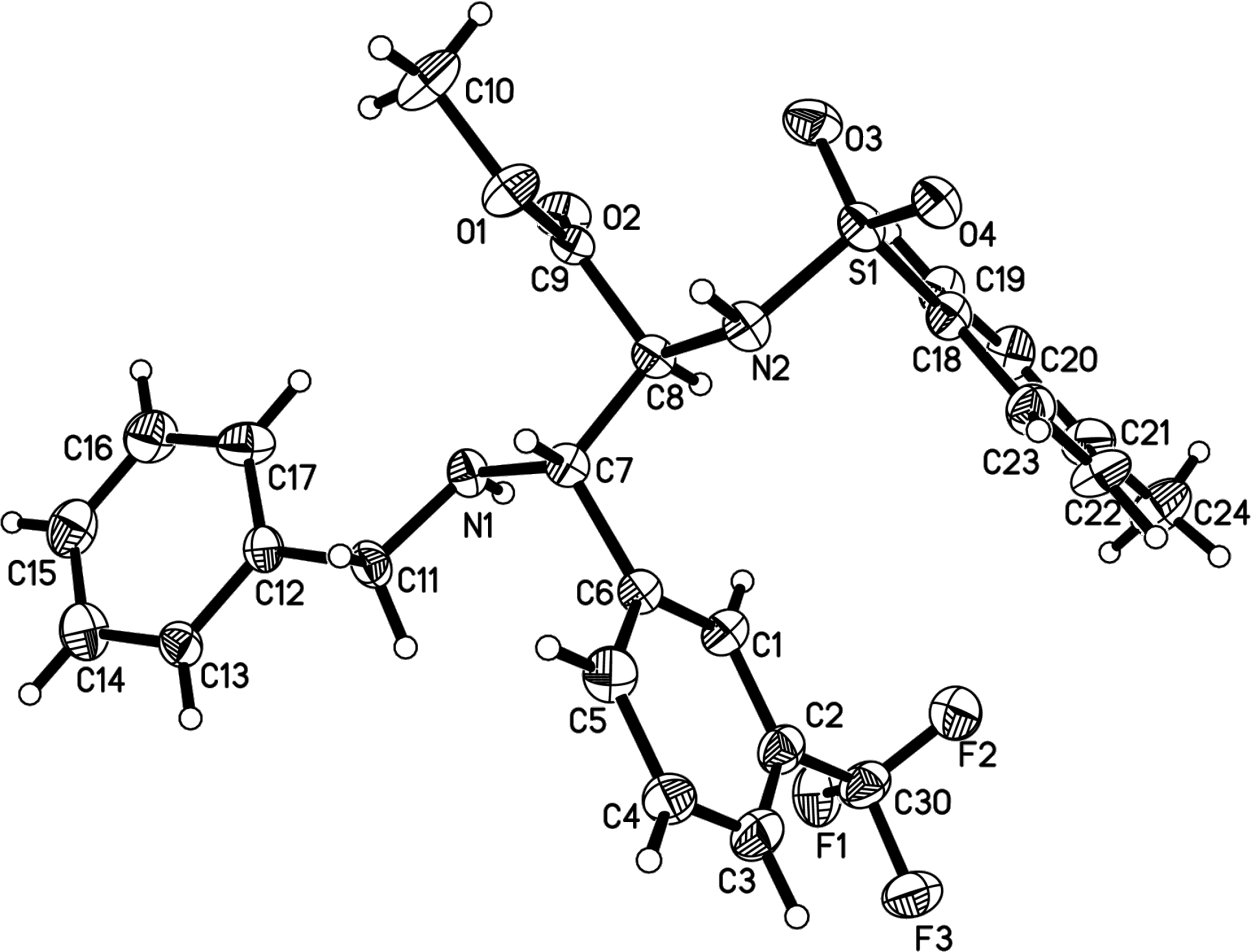
ORTEP diagram of compound **5o**.

To determine the structure of product **5**, single crystals were prepared. Fortunately, the crystals of product **5o** had a good crystallinity and were suitable for single crystal X-ray analysis (Figure 1). Crystallographic analysis has revealed that the *anti*-vicinal diamino ester was obtained. As a result, the stereochemistry of the other products was assigned (*anti*-isomer) based on the similarity of their properties.

**Scheme 3:**
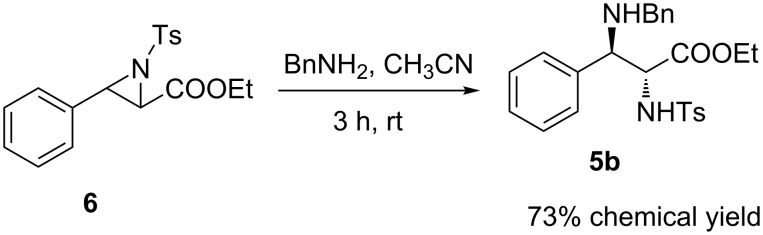
Ring-opening of aziridine **6**.

Finally, some reactions were additionally conducted to gain insight into the reaction mechanism. First, we prepared the aziridine **6** according to the reported method with cinnamic ethyl ester as starting material [33]. Then, we used the aziridine **6** as starting material to react with benzylamine under similar reaction conditions of the third step of this one-pot reaction (Scheme 3). To our delight, aziridine **6** was converted into the corresponding diamino acid ester **5b** with 73% chemical yield. Thus, aziridine most likely might be the intermediate in this reaction.

**Scheme 4:**
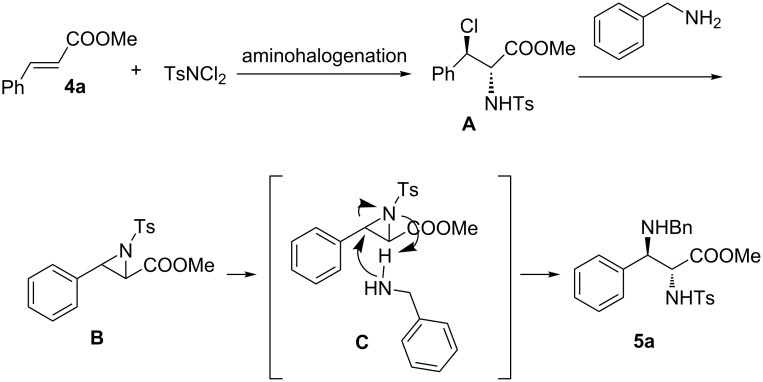
Proposed mechanism.

Based on the above results, a proposed reaction mechanism for this one-pot reaction is illustrated in Scheme 4, which contains the sequence of aminochlorination, aziridination and followed by the S_N_2 nucleophilic ring-opening. The first step is the Cu-catalyzed aminochlorination reaction of methyl cinnamate **1a** resulting in *anti*-chloroamine intermediate **A**. The second step involves a typical intramolecular S_N_2 substitution reaction of intermediate **A** with the aid of benzylamine, to give the aziridine intermediate **B**. The intermediate **B** undergoes a S_N_2 nucleophilic process attacked by benzylamine, leading to the formation of the final product **5a**. The excellent stereoselectivity and formation of only *anti*-isomer can be explained by the formation of aziridine intermediate and complete geometry control of the following S_N_2 nucleophilic attack. The formation of the unexpected diamino ester, instead of aziridine, may be due to the relative strong nucleophilicity of benzylamine. Considering the fact that the final product **5a** is *anti* and the aminohalogenation product intermediate **A** is also *anti*, the only way to explain the stereochemistry of product **5** is the double inversion via aziridine formation. The direct substitution of the Cl atom is possible, but it will lead to the *syn* product **5**. Therefore we believe that the interpretation of the observed stereochemical outcome must involve the intermediate aziridine formation.

## Conclusion

In conclusion, a new one-pot method for the synthesis of α,β-differentiated diamino esters directly from α,β-unsaturated esters has been developed. The reaction sequence includes copper-catalyzed aminochlorination, aziridination and S_N_2 nucleophilic ring-opening reaction. This one-pot reaction is operationally convenient and can tolerate a variety of substrates affording the target products in good-to-excellent chemical yields. Furthermore, this reaction gives virtually complete stereochemical outcomes, and only the *anti*-isomer is found for all the cases, which provides an easy access to α,β-diamino acid derivatives.

## Experimental

**General procedure for the one-pot synthesis of α,β-diamino esters:** Into a dry vial was added cinnamic ester **4** (0.50 mmol) and freshly distilled acetonitrile (3.0 mL). The reaction vial was loaded with freshly activated 4 Å molecular sieves (250 mg), TsNCl_2_ (1.0 mmol) and Cu(OTf)_2_ (10 mol %). The solution in the capped vial was stirred at room temperature for 24 h without argon protection. The reaction was finally quenched by dropwise addition of saturated aqueous Na_2_SO_3_ solution (3.0 mL). After quench for 30 min, benzylamine (2.0 mL) was added to the mixture exposed to air. Another one hour was needed until conversion was complete. Then the phases were separated, and the aqueous phase was extracted with ethyl acetate (3 × 10 mL). The combined organic layers were washed with brine, dried over anhydrous sodium sulfate, and concentrated to dryness. Purification by flash chromatography (EtOAc/hexane, from 1:20 to 1:3, v/v) provided final products **5**.

## Supporting Information

File 1 Experimental details and spectral data.
