# Supplementary material for: One-pot stereoselective synthesis of α,β-differentiated diamino esters via the sequence of aminochlorination, aziridination and intermolecular SN2 reaction
Source: Beilstein J Org Chem. 2014 Aug 7;10:1802–7. doi: 10.3762/bjoc.10.189 (PMC4142878; doi:10.3762/bjoc.10.189)

Supporting Information

for

One-pot stereoselective synthesis of α,β-differentiated diamino esters via the sequence of aminochlorination, aziridination and intermolecular S_N2 reaction

Yiwen Xiong[1], Ping Qian[1], Chenhui Cao[1], Haibo Mei[1], Jianlin Han*[1,2,3], Guigen Li[2,4] and Yi Pan*[1]

Address: [1]School of Chemsitry and Chemical Engineering, State of Key Laboratory of Coordination, Nanjing University, Nanjing, 210093, China, [2]Institute for Chemistry & BioMedical Sciences, Nanjing University, Nanjing, 210093, China, [3]High-Tech Research Institute of Nanjing University, Changzhou, 213164, China and [4]Department of Chemsitry and Biochemistry, Texas Tech Unviersity, Lubbock, Texas, 79409-1061, USA

Email: Jianlin Han* - hanjl@nju.edu.cn; Yi Pan yipan@nju.edu.cn
*Corresponding author

Experimental details and spectral data

Table of Contents

1. General remarks……………………………………………………….……S2

2. General procedure for one-pot synthesis of α,β-diamino esters……S2

3. General procedure for ring-opening of aziridine **6**…………………S8

4. X-ray crystallography for **5o**………………………………………….…S9

5. ^{1}H and ^{13}C NMR spectra for compounds **5**……….…………….…S10

1 General remarks

All the reactions were carried out in oven-dried glassware and all commercially available reagents were used without further purification. Dichloromethane and acetonitrile used for the reaction were distilled using calcium hydride under nitrogen prior to use. ^{1}H and ^{13}C NMR spectra (TMS used as internal standard) were recorded at ambient temperature at 300 MHz, 75 MHz respectively in $CDCl_3$ with a Bruker ARX300 spectrometer. High resolution mass spectra for all the new compounds were done by a Micro mass Q-Tof instrument (ESI). Analytical thin-layer chromatography (TLC) was performed using 0.25 mm precoated silica gel plates and the compounds were visualized with UV light (λ = 254 nm). Compounds were purified using flash column chromatography on silica gel (200–300mesh).

2 General procedure for one-pot synthesis of α,β-diamino esters

Into a dry vial was added cinnamic ester **4** (0.50 mmol) and freshly distilled acetonitrile (3.0 mL). The reaction vial was loaded with freshly activated 4 Å molecular sieves (250 mg), $TsNCl_2$ (1.0 mmol) and $Cu(OTf)_2$ (10 mol %). The solution in the capped vial was stirred at room temperature for 24 h without argon protection. The reaction was finally quenched by dropwise addition of saturated aqueous Na_2SO_3 solution (3.0 mL). After quenching for 30 min, benzylamine (2.0 mL) was added to the mixture exposed to air. Another hour was needed until conversion was complete. Then the phases were separated, and the aqueous phase was extracted with ethyl acetate (3 × 10 mL). The combined organic layers were washed with brine, dried over anhydrous sodium sulfate, and concentrated to dryness. Purification by flash chromatography (EtOAc/hexane, from1:20 to 1:3, v/v) provided final products **5**.

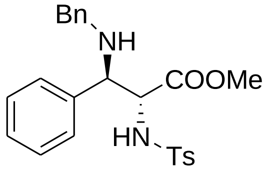

5a Pale yellow oil; ^{1}H NMR (300 MHz, $CDCl_3$) δ 7.64 (d, J = 6.3 Hz, 2H), 7.31-7.20 (m, 10H), 7.08 (dd, J = 5.7, 0.9 Hz, 2H), 5.37 (d, J = 6.6 Hz, 1H), 4.25 (d, J = 5.4 Hz, 1H), 3.98 (d, J = 3.6 Hz, 1H), 3.70 (d, J = 9.6 Hz, 1H), 3.55 (d, J = 9.9 Hz, 1H), 3.36 (s, 3H), 2.38 (s, 3H) ; ^{13}C NMR (75 MHz, $CDCl_3$) δ 170.2,

143.6, 139.4, 137.4 136.6, 129.6, 128.7, 128.4, 128.2, 127.3, 127.2, 62.7, 59.7, 52.1, 50.7, 21.5; HRMS-(ESI) *m/z* $[M+H]^+$ calcd for $C_{24}H_{27}N_2O_4S$, 439.1692; found, 439.1694

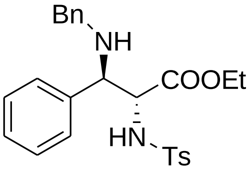

5b Pale yellow oil; ^{1}H NMR (300 MHz, $CDCl_3$) δ 7.79 (d, J = 8.4 Hz, 2H), 7.34-7.23 (m, 10H), 7.14 (dd, J = 7.5, 2.1 Hz, 2H), 5.38 (d, J = 5.7 Hz, 1H), 4.29 (s, 1H), 4.05 (d, J = 4.8 Hz, 1H), 3.82 (qd, J = 7.2, 1.2 Hz, 2H) , 3.75 (d, J = 13.2 Hz, 1H), 3.61 (d, J = 12.9 Hz, 1H), 2.40 (s, 3H) , 0.98 (t, J = 6.9 Hz, 3H) ; ^{13}C NMR (75 MHz, $CDCl_3$) δ 169.6, 143.6, 139.4, 137.4, 136.6, 129.6, 128.7, 128.4, 128.3, 128.2, 127.3, 127.1, 62.7, 61.5, 59.6, 50.7, 21.5, 13.8; HRMS-(ESI) *m/z* $[M+H]^+$ calcd for $C_{25}H_{29}N_2O_4S$, 453.1848; found, 453.1860

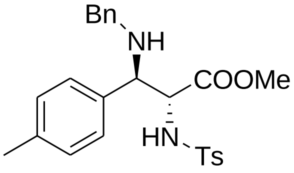

5c Pale yellow oil; ^{1}H NMR (300 MHz, $CDCl_3$) δ 7.68 (d, J = 8.1 Hz, 2H), 7.34-7.23 (m, 7H), 7.15 (d, J =7.8 Hz, 2H), 7.00 (d, J =8.1 Hz, 2H), 5.41 (br, 1H), 4.28 (s, 1H), 3.98 (d, J = 5.1 Hz, 1H), 3.73 (d, J = 13.2 Hz, 1H), 3.58 (d, J = 13.2 Hz, 1H), 3.41 (s, 3H), 2.41 (s, 3H) , 2.36 (s, 3H) ; ^{13}C NMR (75 MHz, $CDCl_3$) δ 170.3, 143.6, 139.5, 137.9, 136.6, 134.3, 129.5, 129.4, 128.4, 128.3, 127.3, 127.1, 62.4, 59.8, 52.1, 50.7, 21.5, 21.1; HRMS-(ESI) *m/z* $[M+H]^+$ calcd for $C_{25}H_{29}N_2O_4S$, 453.1848; found, 453.1840

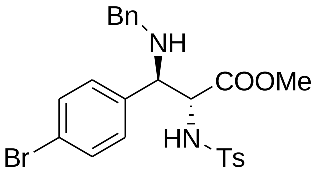

5d Pale yellow oil; ^{1}H NMR (300 MHz, $CDCl_3$) δ 7.65 (d, J = 8.1 Hz, 2H), 7.42 (d, J = 8.1 Hz, 2H), 7.33-7.23 (m, 7H), 7.01 (d, J =8.4 Hz, 2H), 5.43 (d, J =6.9 Hz, 1H), 4.25 (s, 1H), 3.97 (d, J = 5.1 Hz, 1H), 3.71 (d, J = 13.2 Hz, 1H), 3.56 (d, J = 13.2 Hz, 1H), 3.44 (s, 3H), 2.42 (s, 3H) ; ^{13}C NMR (75 MHz, $CDCl_3$) δ 170.2, 143.8, 139.2, 136.8, 136.5, 131.8, 129.6, 129.0, 128.4, 128.2, 127.2,

122.1, 62.3, 59.6, 52.3, 50.7, 21.6; HRMS-(ESI) *m/z* $[M+H]^+$ calcd for $C_{24}H_{26}BrN_2O_4S$, 517.0797; found, 517.0793

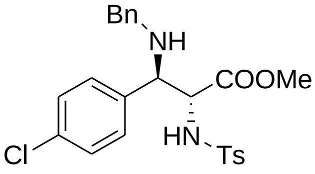

5e Pale yellow oil; ^1H NMR (300 MHz, $CDCl_3$) δ 7.65 (dd, *J* = 8.1,1.8 Hz, 2H), 7.33-7.24 (m, 9H), 7.07 (dd, *J* =8.1, 1.8 Hz, 2H), 5.37 (d, *J* =3.3 Hz, 1H), 4.26 (s, 1H), 3.99 (d, *J* = 2.1 Hz, 1H), 3.71 (d, *J* = 13.2 Hz, 1H), 3.57 (d, *J* = 11.8 Hz, 1H), 3.43 (s, 3H), 2.42 (s, 3H); ^{13}C NMR (75 MHz, $CDCl_3$) δ 170.3, 143.8, 139.3, 136.3, 134.1, 129.7, 128.9, 128.8, 128.5, 127.3, 62.3, 59.8, 52.4, 50.8, 21.6; HRMS-(ESI) *m/z* $[M+H]^+$ calcd for $C_{24}H_{26}ClN_2O_4S$, 473.1302; found, 473.1300

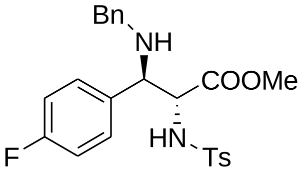

5f Pale yellow oil; ^1H NMR (300 MHz, $CDCl_3$) δ 7.67 (d, *J* = 8.1 Hz, 2H), 7.33-6.98 (m, 11H), 5.42 (br, 1H), 4.27 (d, *J* = 4.5 Hz, 1H), 4.01 (d, *J* = 5.1 Hz, 1H), 3.72 (d, *J* = 13.2 Hz, 1H), 3.57 (d, *J* = 13.2 Hz, 1H), 3.42 (s, 3H), 2.42 (s, 3H); ^{13}C NMR (75 MHz, $CDCl_3$) δ 170.2, 164.1, 160.8, 143.7, 139.3, 136.6, 133.4, 129.6, 129.0, 128.9, 128.4, 128.2, 127.3, 127.2, 115.6, 62.1, 59.8, 52.2, 50.7, 21.5; HRMS-(ESI) *m/z* $[M+H]^+$ calcd for $C_{24}H_{26}FN_2O_4S$, 457.1597; found, 457.1601

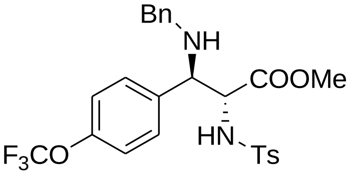

5g Pale yellow oil; ^1H NMR (300 MHz, $CDCl_3$) δ 7.67 (d, *J* = 8.4 Hz, 2H), 7.33-7.17 (m, 11H), 5.50 (br, 1H), 4.28 (s, 1H), 4.04 (d, *J* = 5.1 Hz, 1H), 3.72 (d, *J* = 13.2 Hz, 1H), 3.58 (d, *J* = 12.9 Hz, 1H), 3.42 (s, 3H), 2.41 (s, 3H); ^{13}C NMR (75 MHz, $CDCl_3$) δ 170.2, 148.9, 143.8, 139.3, 136.6, 129.6, 128.8, 128.4, 128.2, 127.2, 121.0, 62.2, 59.8, 52.2, 50.8, 21.4; HRMS-(ESI) *m/z* $[M+H]^+$ calcd for $C_{25}H_{26}F_3N_2O_5S$, 523.1515; found, 523.1495

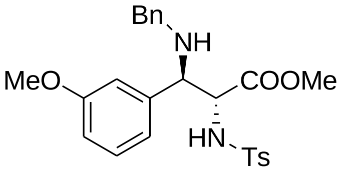

5h Pale yellow oil; ^{1}H NMR (300 MHz, $CDCl_3$) δ 7.63 (d, J = 8.4 Hz, 2H), 7.34-7.20 (m, 9H), 6.80-6.77 (m, 2H), 5.71 (br, 1H), 4.33-4.26 (m, 2H), 3.77-3.67 (m, 4H), 3.48-3.43 (m, 4H), 2.41 (s, 3H); ^{13}C NMR (75 MHz, $CDCl_3$) δ 170.4, 158.6, 143.6 , 139.2, 136.6, 135.9, 130.6, 129.6, 128.5, 128.4, 127.3, 114.8, 113.4, 59.3, 58.0, 55.6, 52.2, 50.8, 21.5; HRMS-(ESI) *m/z* $[M+Na]^+$ calcd for $C_{25}H_{28}N_2O_5S$ Na, 491.1617; found, 491.1674

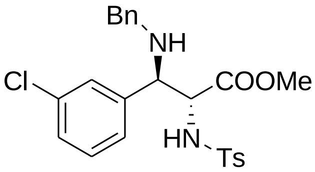

5i Pale yellow oil; ^{1}H NMR (300 MHz, $CDCl_3$) δ 7.67 (d, J = 8.1 Hz, 2H), 7.34-7.24 (m, 9H), 7.09-7.03 (m, 2H), 5.41 (d, J = 9.3 Hz, 1H), 4.26 (dd, J = 8.7, 4.8 Hz, 2H), 3.73 (d, J = 13.2 Hz, 1H), 3.58 (d, J = 13.2 Hz, 1H), 3.44 (s, 3H) 2.42 (s, 3H); ^{13}C NMR (75 MHz, $CDCl_3$) δ 170.1, 143.8, 139.9, 139.1, 136.4, 134.7,130.0, 129.6, 128.4, 128.2, 127.4, 127.2, 126.4, 125.5, 62.4, 125.5, 62.4, 59.7, 52.3, 50.7, 21.5; HRMS-(ESI) *m/z* $[M+H]^+$ calcd for $C_{24}H_{26}ClN_2O_4S$, 473.1302; found, 473.1296

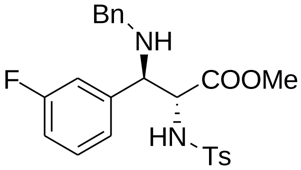

5j Pale yellow oil; ^{1}H NMR (300 MHz, $CDCl_3$) δ 7.68 (d, J = 8.1 Hz, 2H), 7.34-7.23 (m, 8H), 6.99 (td, J = 8.4, 2.1 Hz, 1H), 6.92 (d, J = 7.8 Hz, 1H), 6.85 (d, J = 9.6 Hz, 1H), 5.46 (br, 1H), 4.28 (d, J = 4.2 Hz, 1H), 4.01 (d, J = 5.1 Hz, 1H), 3.73 (d, J = 13.2 Hz, 1H), 3.58 (d, J = 13.2 Hz, 1H), 3.43 (s, 3H), 2.41 (s, 3H); ^{13}C NMR (75 MHz, $CDCl_3$) δ 170.2, 164.2, 161.8, 143.8, 139.3, 136.5, 130.3, 129.6, 128.4, 128.2, 127.3, 127.2, 123.1, 115.1, 114.2, 62.4, 59.7, 52.3, 50.7, 21.5; HRMS-(ESI) *m/z* $[M+H]^+$ calcd for $C_{24}H_{26}FN_2O_4S$, 457.1597; found, 457.1615

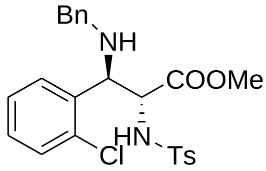

5k Pale yellow oil; ^{1}H NMR (300 MHz, $CDCl_3$) δ 7.64 (d, J = 8.1 Hz, 2H), 7.35-7.21 (m, 11H), 5.70 (br, 1H), 4.38-4.29 (m, 2H), 3.69 (d, J = 12.9 Hz, 1H), 3.45 (d, J = 13.2 Hz, 1H), 3.38 (s, 3H), 2.41 (s, 3H); ^{13}C NMR (75 MHz, $CDCl_3$) δ 170.3, 143.6, 139.1, 136.6, 134.7, 134.2, 130.1, 129.6, 129.2, 128.5, 128.4, 127.8, 127.3, 127.0, 59.0, 57.8, 52.1, 50.7, 21.5; HRMS-(ESI) *m/z* $[M+H]^+$ calcd for $C_{24}H_{26}ClN_2O_4S$, 473.1302; found, 473.1294

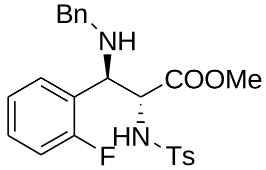

5l Pale yellow oil; ^{1}H NMR (300 MHz, $CDCl_3$) δ 7.67 (d, J = 8.4 Hz, 2H), 7.36-7.16 (m, 10H), 7.01 (t, J = 6.0 Hz, 1H), 5.49 (d, J = 9.0 Hz, 1H), 4.35-4.26 (m, 2H), 3.73 (d, J = 12.9 Hz, 1H), 3.57 (d, J = 12.9 Hz, 1H), 3.44 (s, 3H), 2.41 (s, 3H); ^{13}C NMR (75 MHz, $CDCl_3$) δ 170.3, 162.6, 159.3, 143.6, 139.3, 136.6, 129.6, 128.6, 128.4, 128.3, 127.3, 124.4, 115.8, 115.5, 58.7, 57.0, 52.2, 51.0, 21.5 ; HRMS-(ESI) *m/z* $[M+H]^+$ calcd for $C_{24}H_{26}FN_2O_4S$, 457.1597; found, 457.1615.

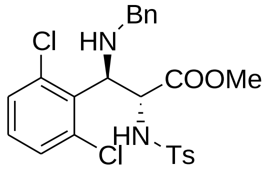

5m Pale yellow oil; ^{1}H NMR (300 MHz, $CDCl_3$) δ 7.52 (d, J = 8.4 Hz, 2H), 7.31-7.13 (m, 10H), 5.30 (d, J = 10.8 Hz, 1H), 4.71 (d, J = 10.8 Hz, 1H), 4.58 (d, J = 10.8 Hz, 1H) 3.61 (d, J = 13.2 Hz, 1H), 3.60 (s, 3H), 3.45 (d, J = 13.2 Hz, 1H), 2.40 (s, 3H); ^{13}C NMR (75 MHz, $CDCl_3$) δ 171.4, 143.4, 139.2, 137.6, 136.4, 133.9, 132.6, 130.0, 129.4, 128.2, 128.1, 127.2, 60.9, 57.9, 52.3, 51.4, 21.5; HRMS-(ESI) *m/z* $[M+H]^+$ calcd for $C_{24}H_{25}Cl_2N_2O_4S$, 507.0912; found, 507.0931

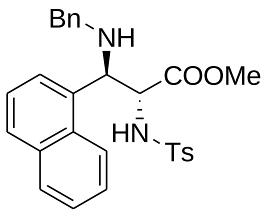

5n Red-brown oil; ^{1}H NMR (300 MHz, $CDCl_3$) δ 7.90 (d, J = 7.5 Hz, 1H), 7.83 (d, J = 7.8 Hz, 1H), 7.64 (d, J = 8.1 Hz, 2H), 7.57 (t, J = 7.5 Hz, 1H), 7.52-7.21 (m, 11H), 5.98 (br, 1H), 4.78 (d, J = 4.8 Hz, 1H), 4.34 (d, J = 4.5 Hz, 1H), 3.83 (d, J = 12.9 Hz, 1H), 3.54 (d, J = 13.2 Hz, 1H), 3.22 (s, 3H), 2.43 (s, 3H) ; ^{13}C NMR (75 MHz, $CDCl_3$) δ 170.2, 143.6, 139.5, 136.7, 134.0, 132.8, 131.5, 129.6, 129.2, 128.7, 128.5, 127.4, 127.3, 126.5, 125.8, 125.1, 123.5, 121.9, 58.5, 57.9, 51.8, 50.8, 21.6; HRMS-(ESI) *m/z* $[M+H]^+$ calcd for $C_{28}H_{29}N_2O_4S$, 489.1848; found, 489.1843

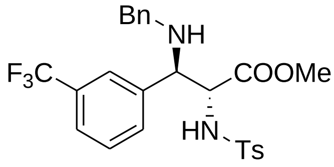

5o white solid; m.p. 118-120 ^oC; ^{1}H NMR (400 MHz, $CDCl_3$) δ 7.64 (d, J = 8.4 Hz, 2H), 7.54 (d, J = 7.6 Hz, 1H), 7.45 (t, J = 7.6 Hz, 1H), 7.37 (d, J = 8.0 Hz, 1H), 7.31-7.21 (m, 8H), 5.34 (d, J = 8.8 Hz, 1H), 4.28-4.24 (m, 1H), 3.70 (d, J = 13.2 Hz, 1H), 3.57 (d, J = 13.2 Hz, 1H), 3.40 (s, 3H), 2.38 (s, 3H); ^{13}C NMR (100 MHz, $CDCl_3$) δ 170.0, 143.9, 139.1 (d, J = 9.9 Hz), 136.4, 131.8, 130.7, 129.6, 129.2, 128.5, 128.2, 127.3 (d, J = 2.7 Hz), 125.0 (d, J = 3.7 Hz), 124.1 (d, J = 3.8 Hz), 62.6, 59.6, 52.3, 50.8, 21.5; HRMS-(ESI) *m/z* $[M+H]^+$ calcd for $C_{25}H_{26}F_3N_2O_4S$, 507.1565; found, 507.1564

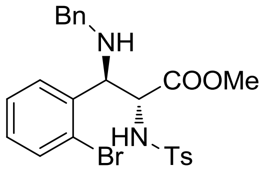

5p Pale yellow oil; ^{1}H NMR (300 MHz, $CDCl_3$) δ 7.64 (d, J = 8.1 Hz, 2H), 7.54 (d, J = 7.8 Hz, 1H), 7.38-7.15 (m, 10H), 5.80 (br, 1H), 4.36-4.26 (m, 2H), 3.69 (d, J = 12.9 Hz, 1H), 3.43 (d, J = 12.9 Hz, 1H), 3.37 (s, 3H), 2.41 (s, 3H); ^{13}C NMR (75 MHz, $CDCl_3$) δ 170.3, 143.6, 139.1, 136.7, 136.4, 133.4, 129.6, 128.5, 128.4, 127.9, 127.6, 127.4, 127.3, 127.0, 124.6, 61.4, 57.8, 52.1, 50.7, 21.5; HRMS-(ESI) *m/z* $[M+H]^+$ calcd for $C_{24}H_{26}BrN_2O_4S$, 517.0797; found, 517.0794.

3 General procedure for ring-opening of aziridine 6

Into a solution of prepared aziridine **6** (0.5 mmol) in dry CH_3CN (4.0 mL) was added benzylamine (5.0 mmol) in an ice bath. Then the temperature was raised to room temperature and the reaction mixture was maintained at rt for another 3 h, resulting in complete conversion. After being concentrated under reduced pressure, the residue was purified via column chromatography with ethyl acetate and petroleum ether (from 1:10 to 1:3 v/v) as eluent to give compound **5b**.

4 X-ray crystallography for 5o

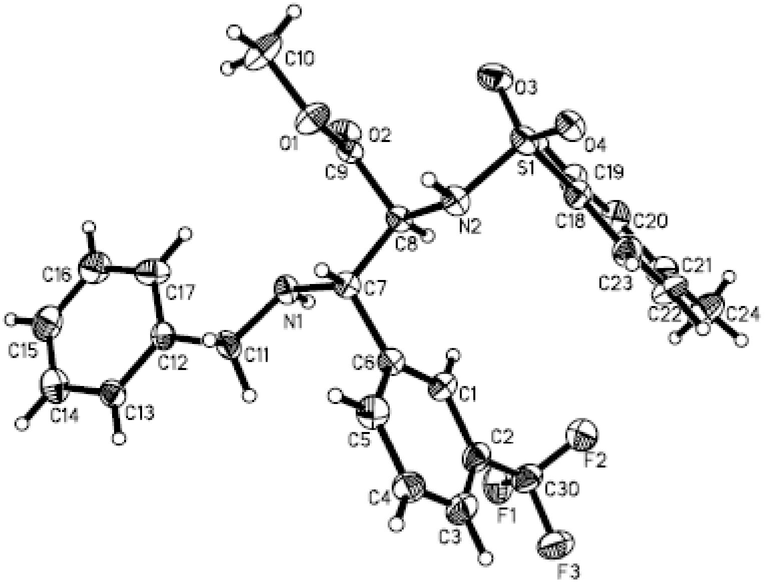

Figure S1: ORTEP diagram of compound **5o** (CCDC 982288).

5 ^{1}H and ^{13}C NMR spectra for compound 5

^{1}H and ^{13}C NMR of **5a** ($CDCl_3$)

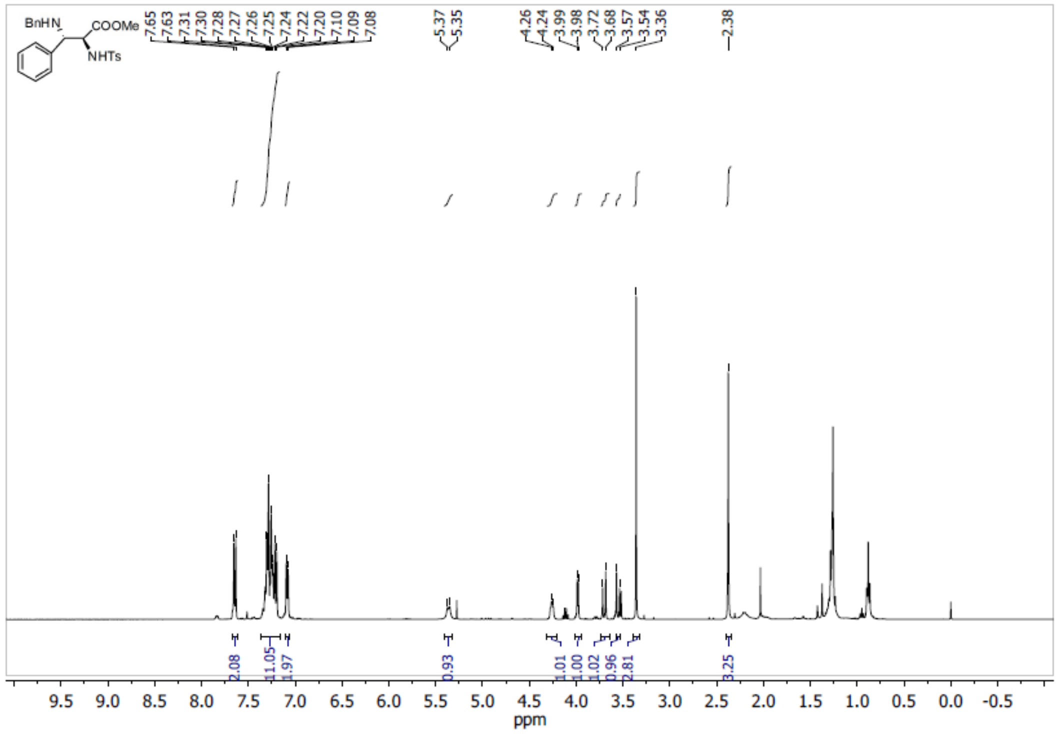

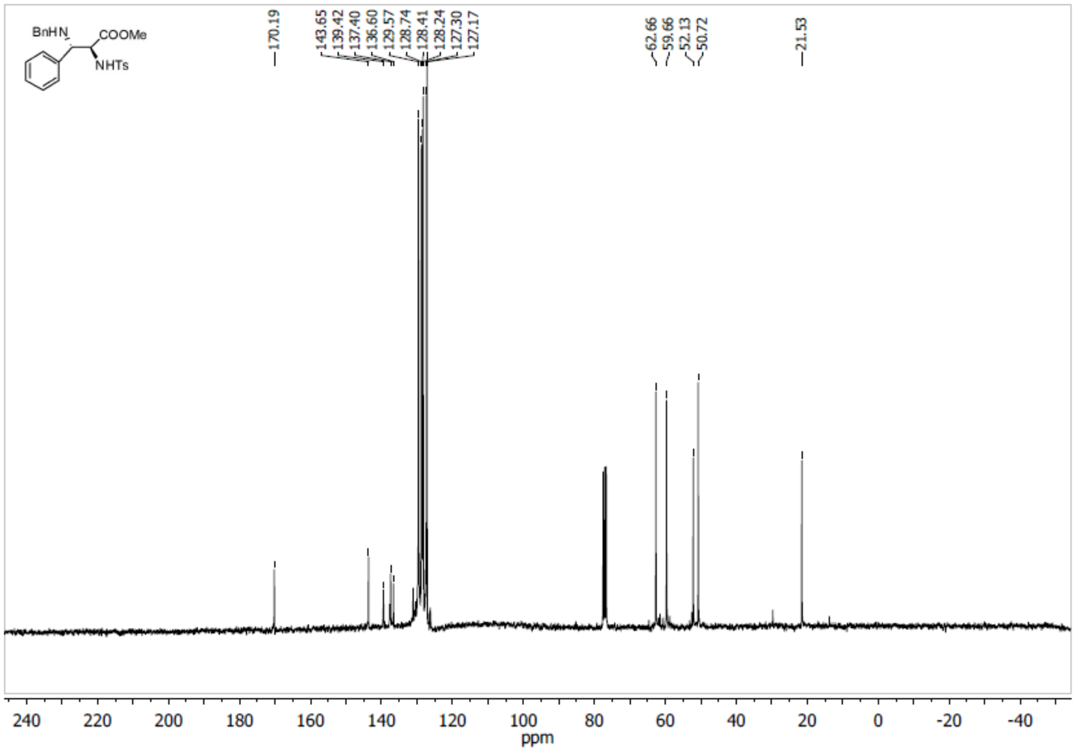

^{1}H and ^{13}C NMR of **5b** ($CDCl_3$)

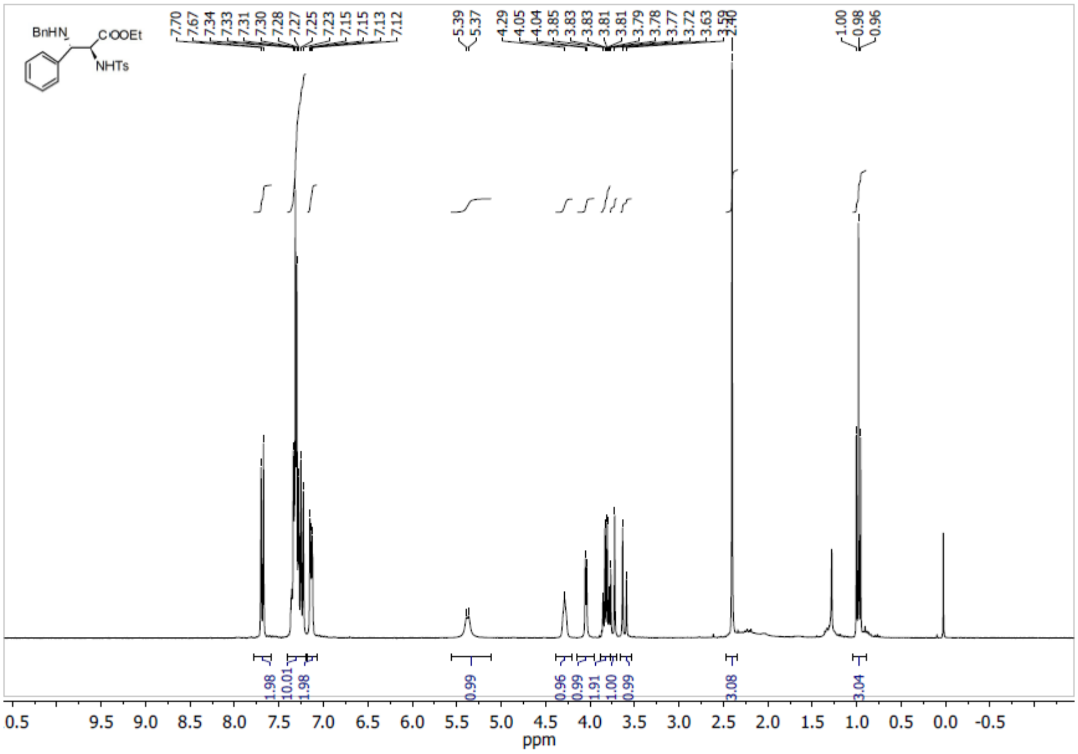

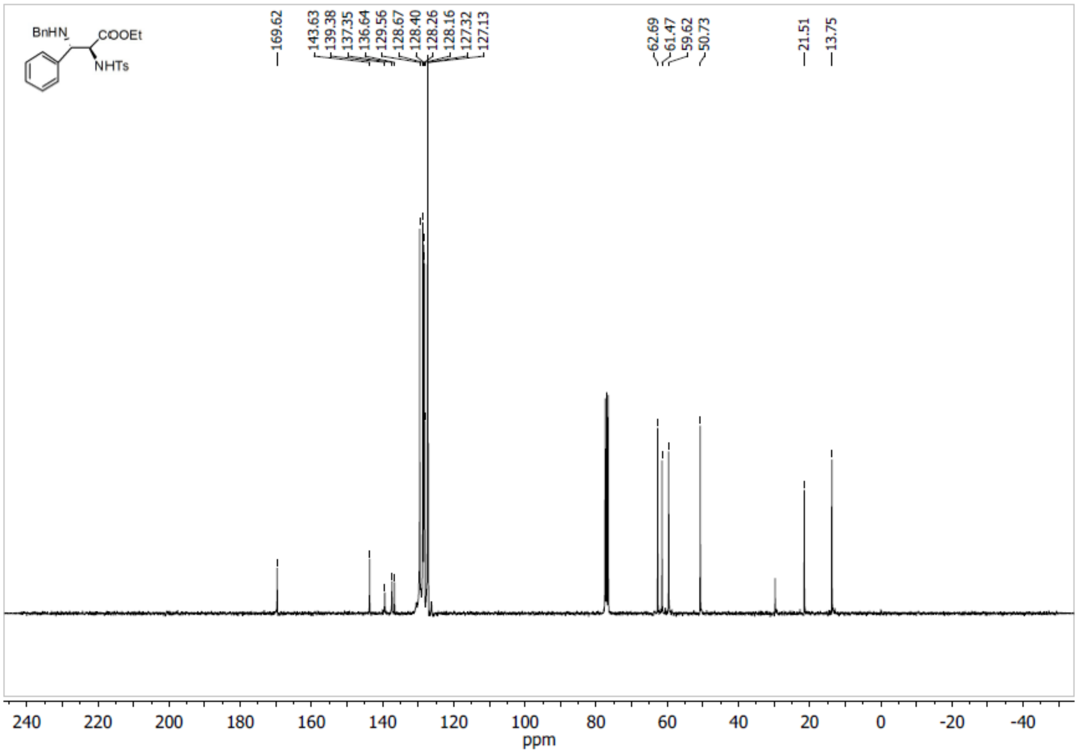

^{1}H and ^{13}C NMR of **5c** ($CDCl_3$)

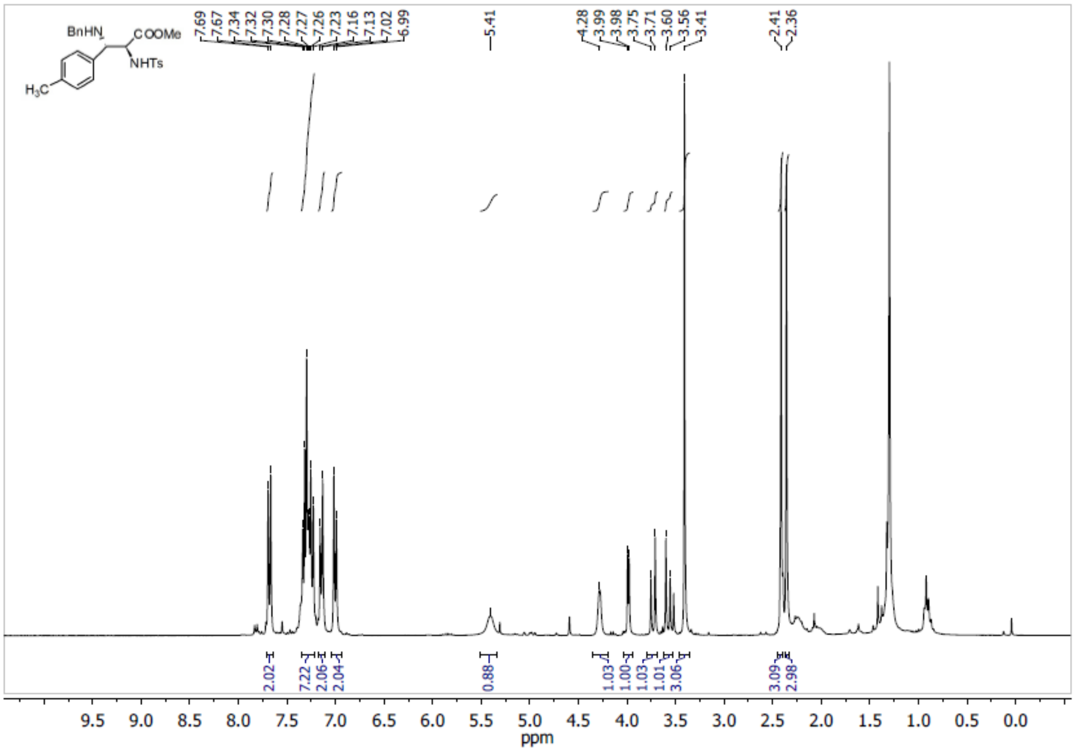

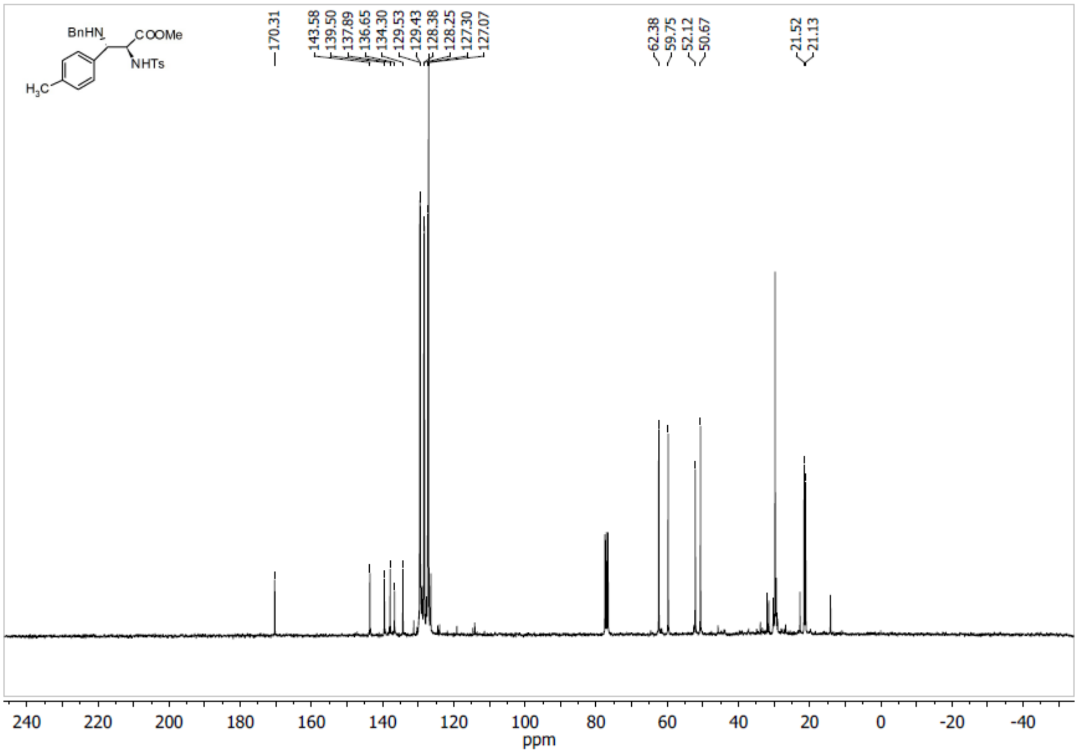

^{1}H and ^{13}C NMR of **5d** ($CDCl_3$)

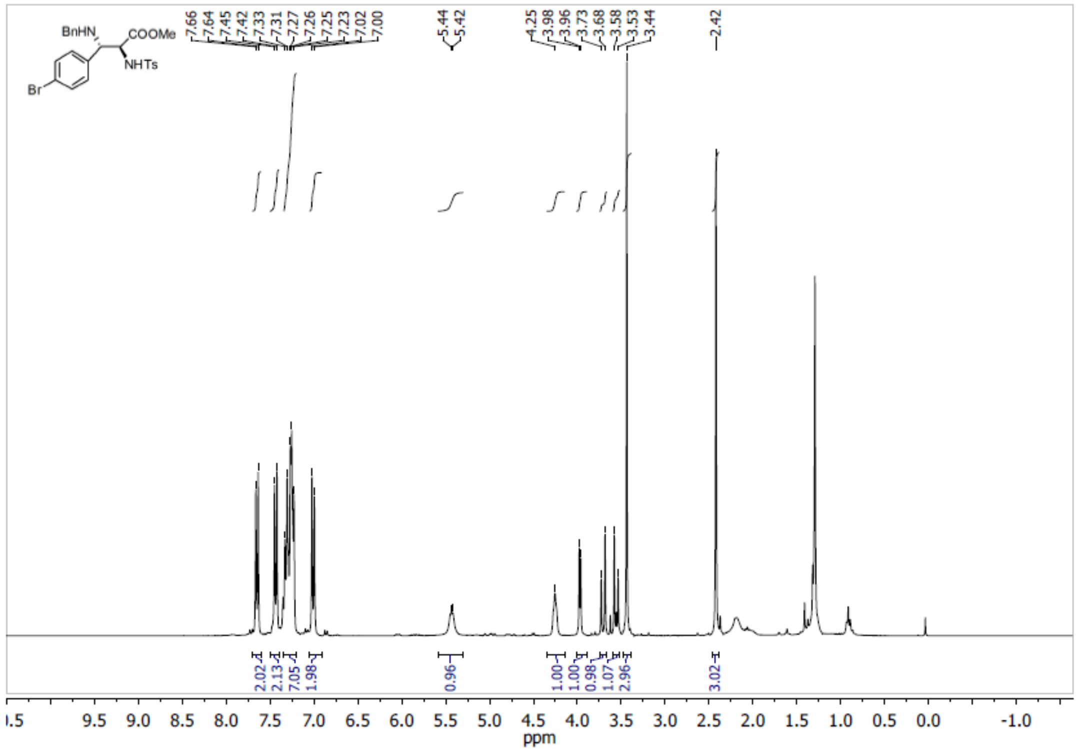

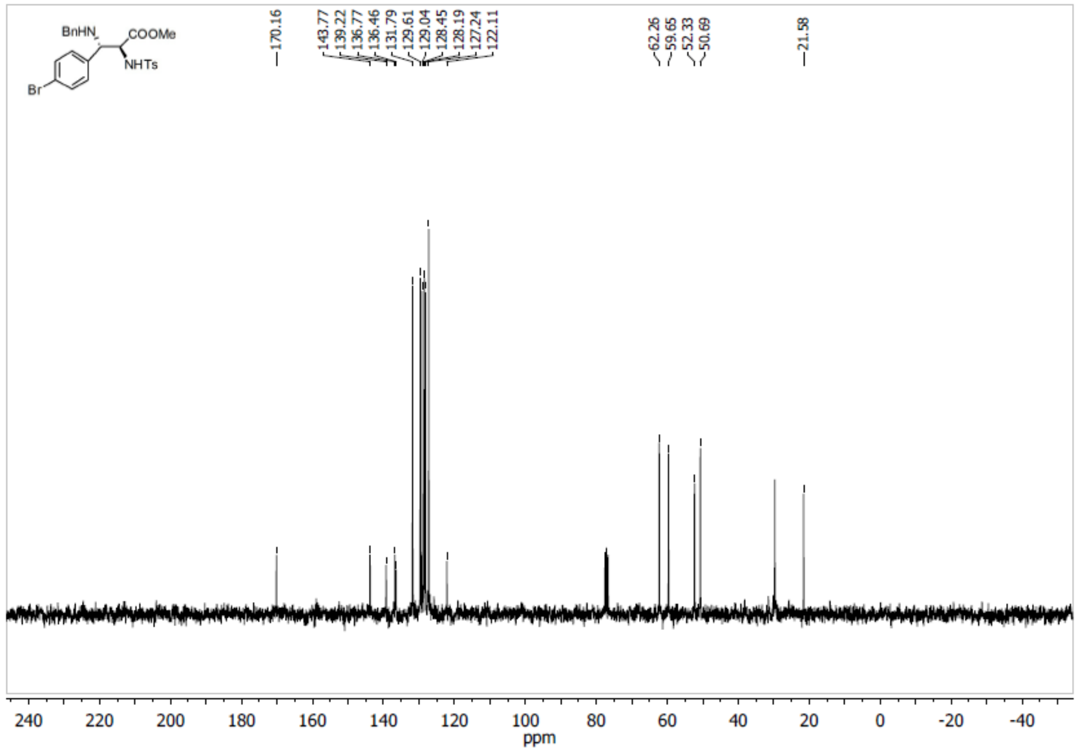

^{1}H and ^{13}C NMR of **5e** ($CDCl_3$)

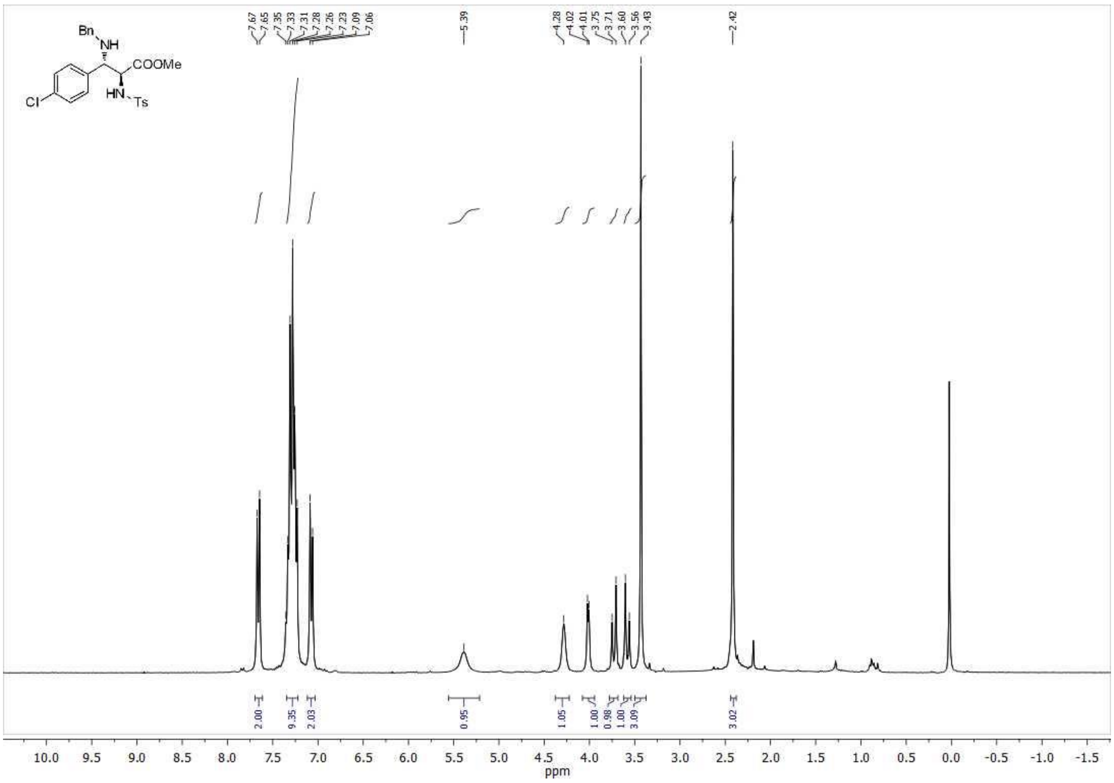

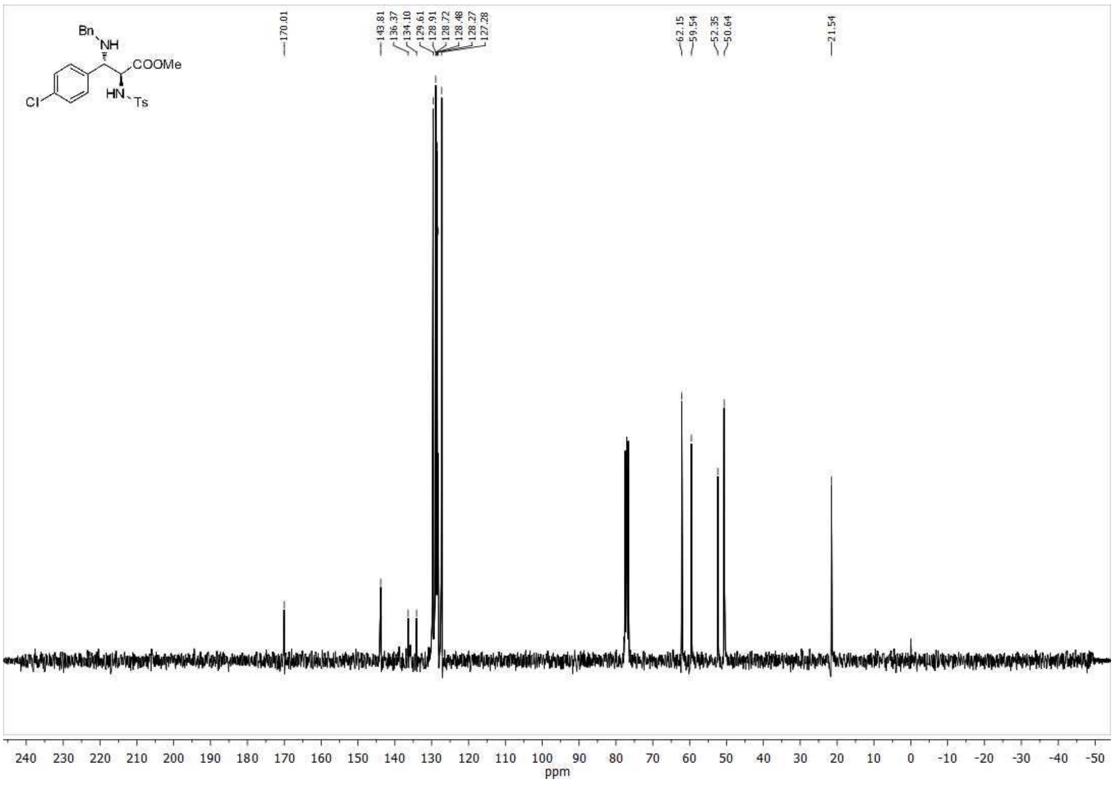

^{1}H and ^{13}C NMR of **5f** ($CDCl_3$)

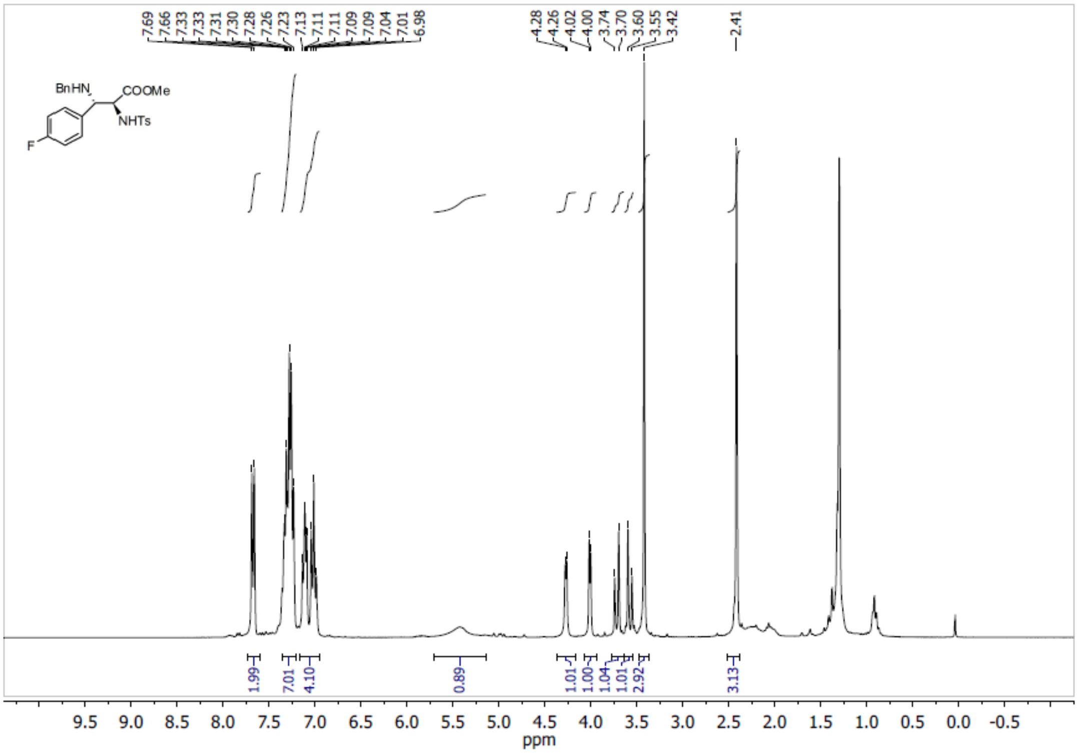

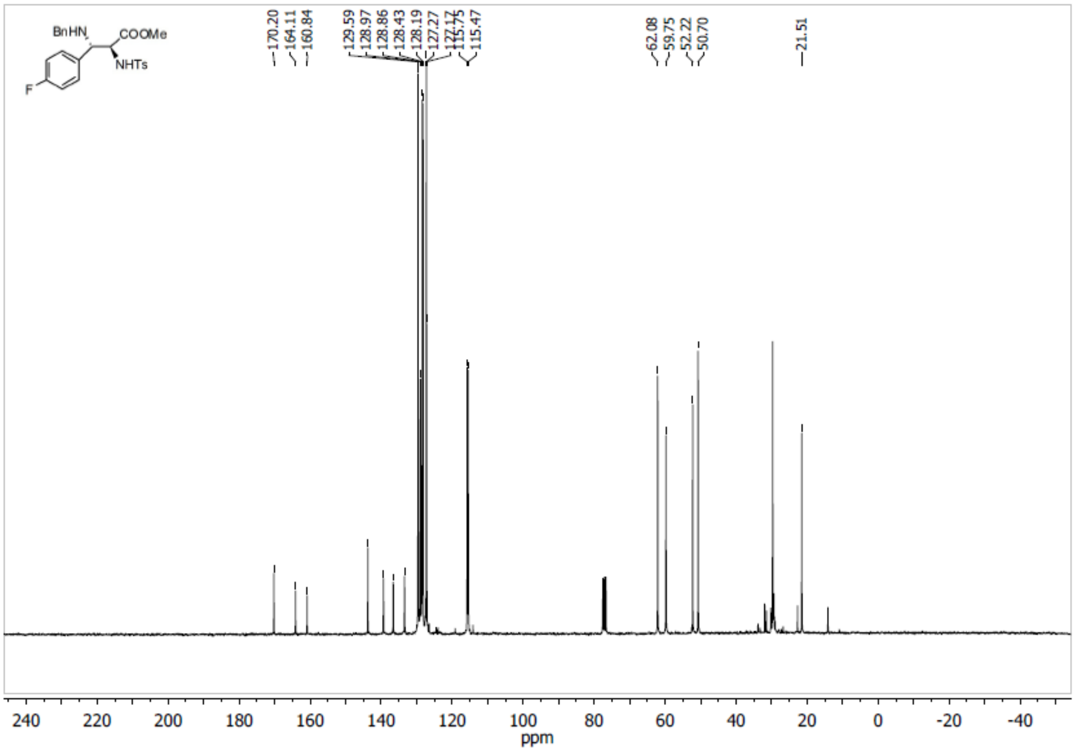

^{1}H and ^{13}C NMR of **5g** ($CDCl_3$)

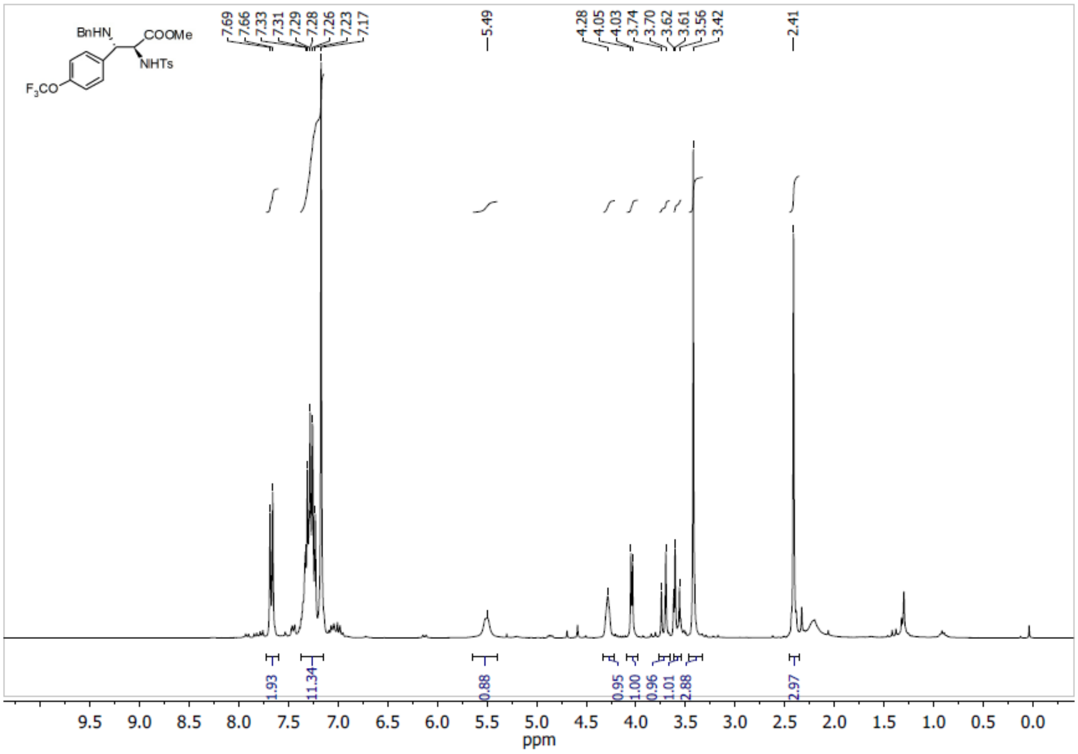

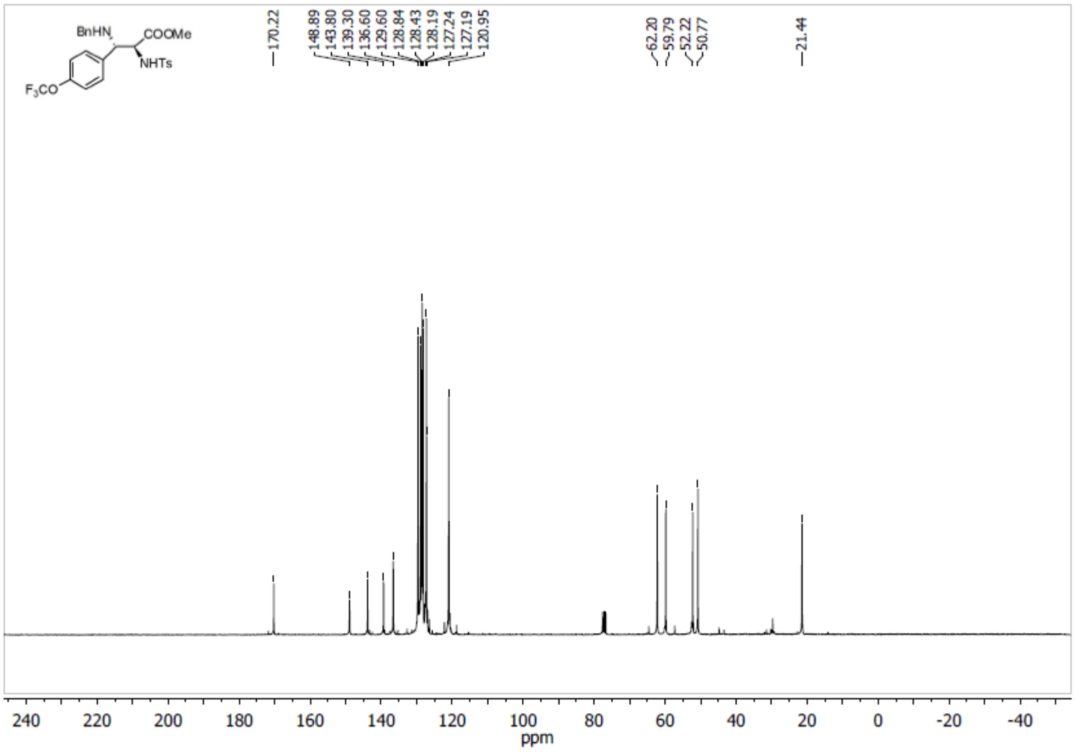

^{1}H and ^{13}C NMR of **5h** ($CDCl_3$)

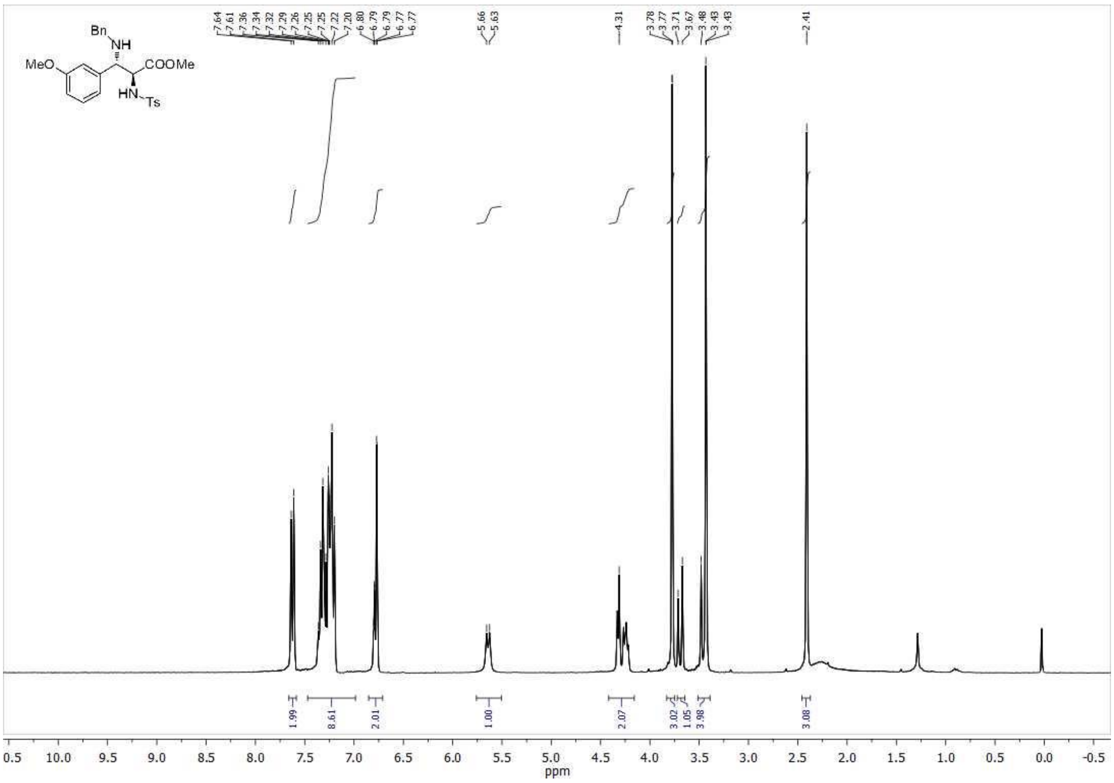

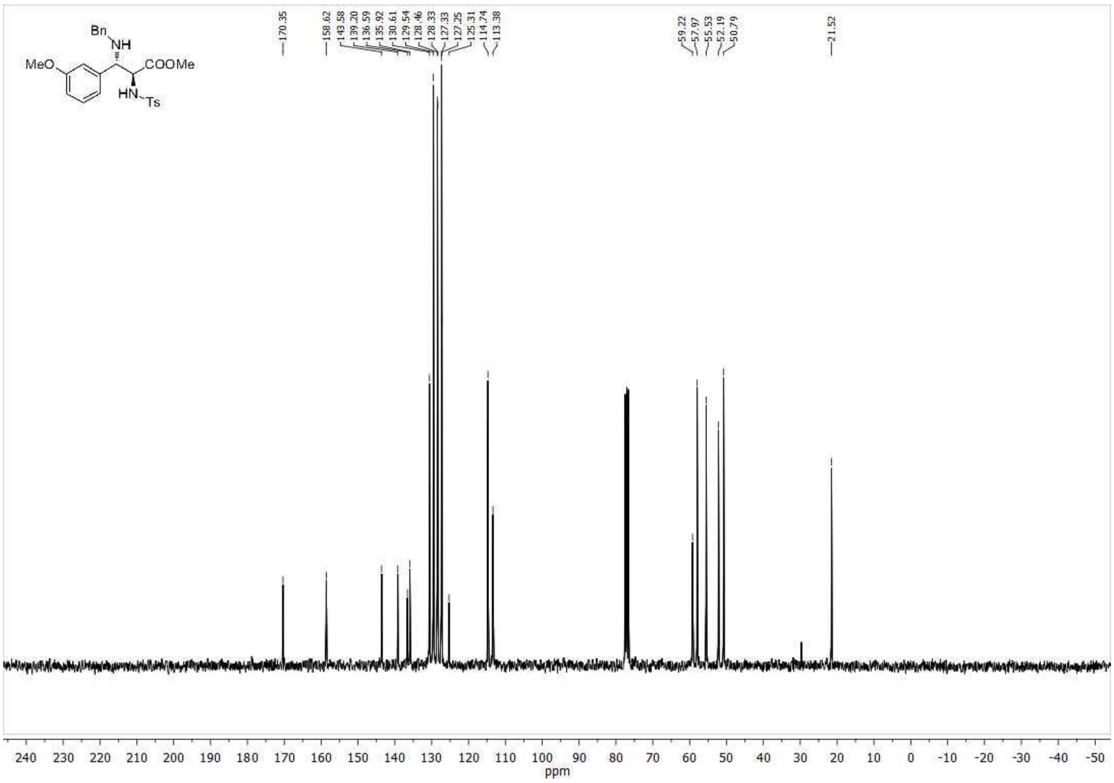

^{1}H and ^{13}C NMR of **5i** ($CDCl_3$)

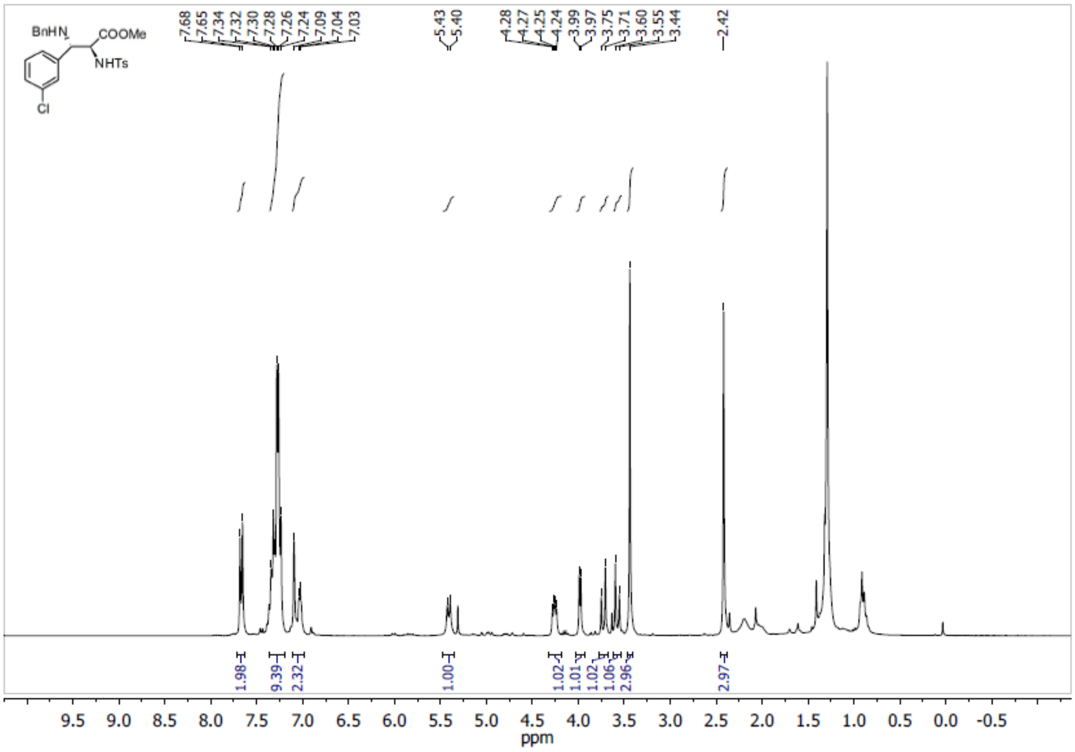

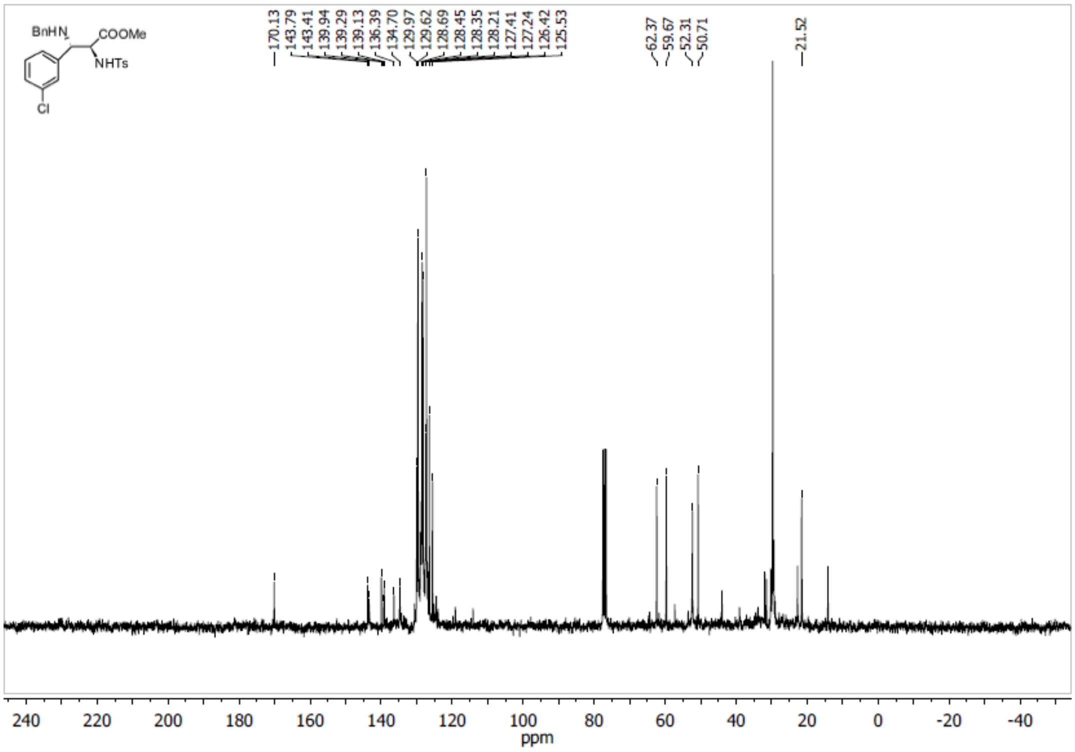

^1H and ^{13}C NMR of **5j** ($CDCl_3$)

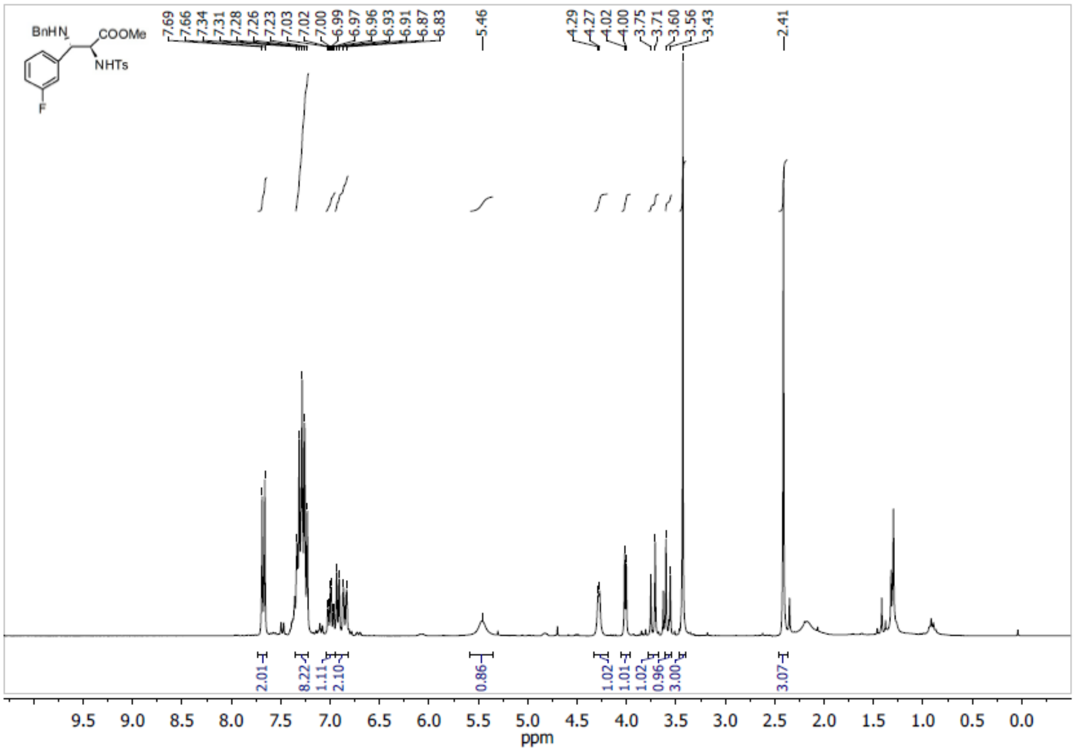

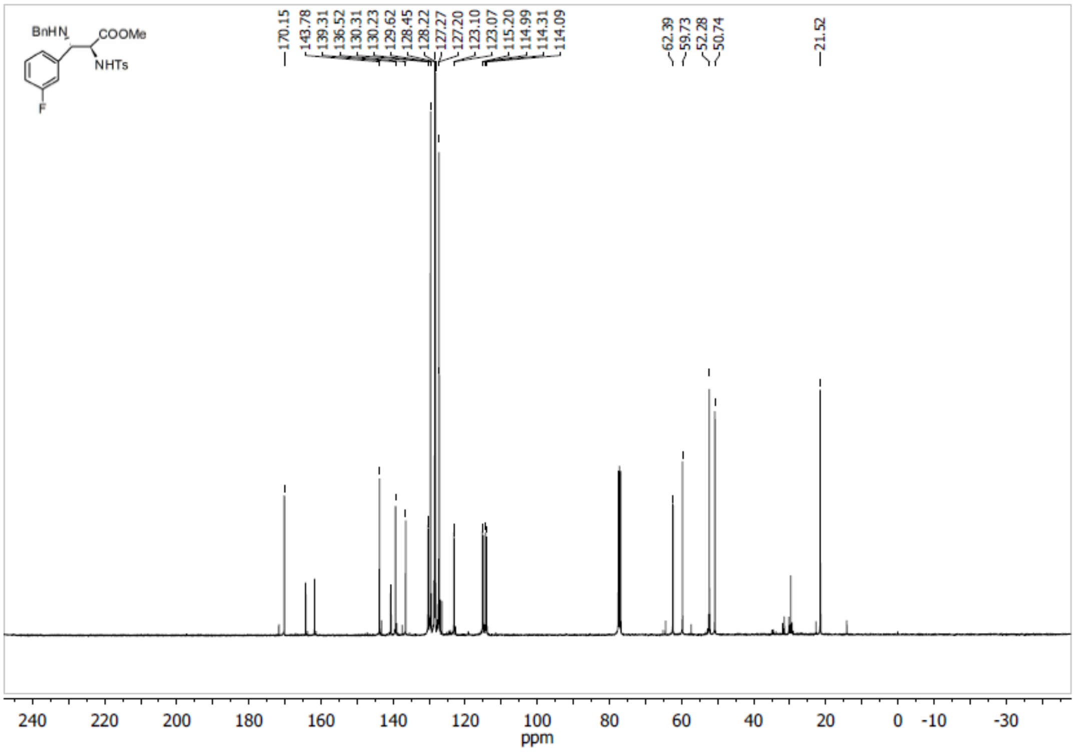

^{1}H and ^{13}C NMR of **5k** ($CDCl_3$)

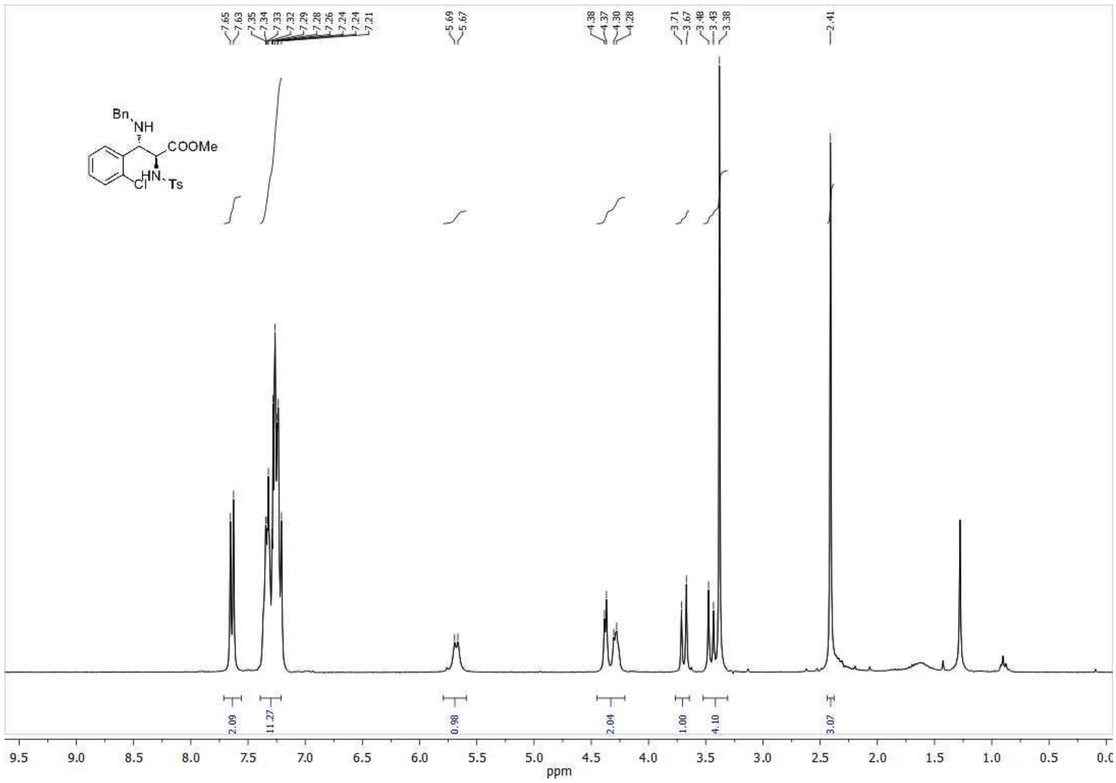

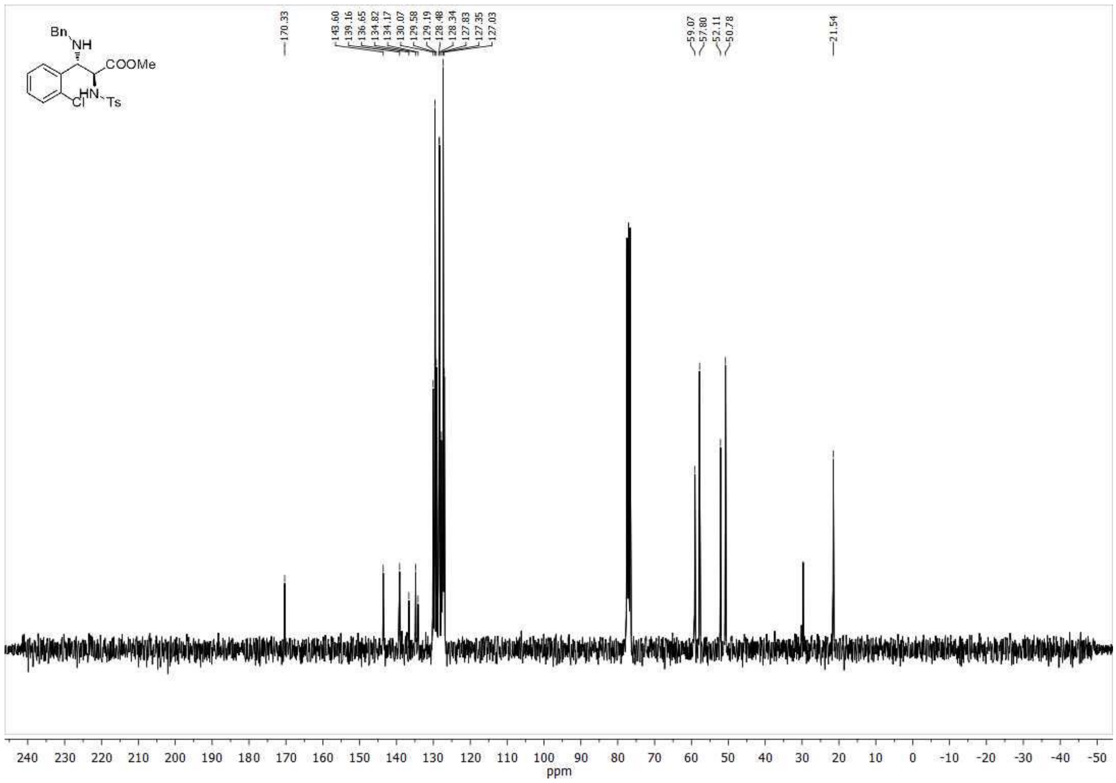

^{1}H and ^{13}C NMR of **5l** ($CDCl_3$)

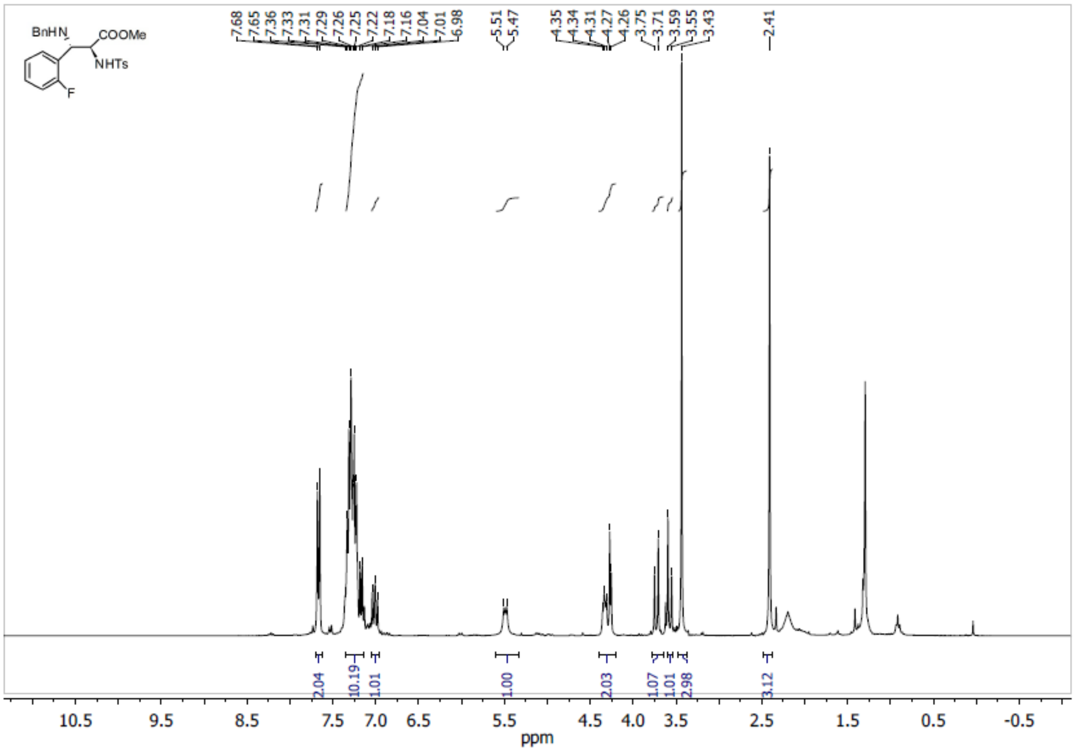

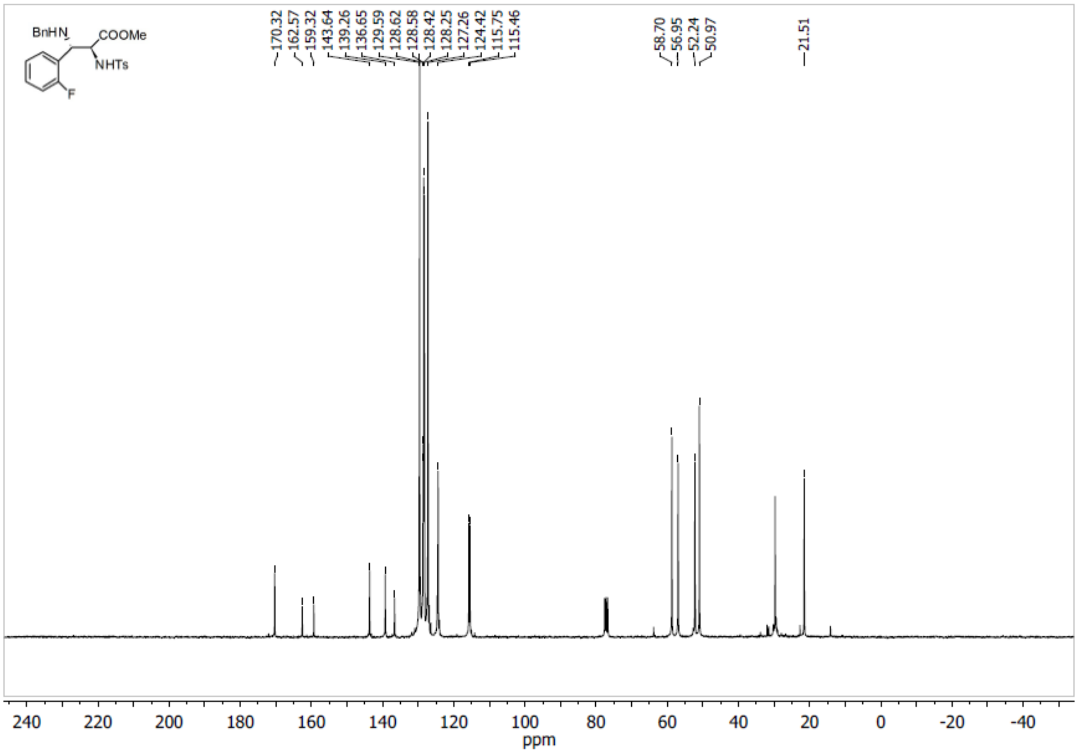

^{1}H and ^{13}C NMR of **5m** ($CDCl_3$)

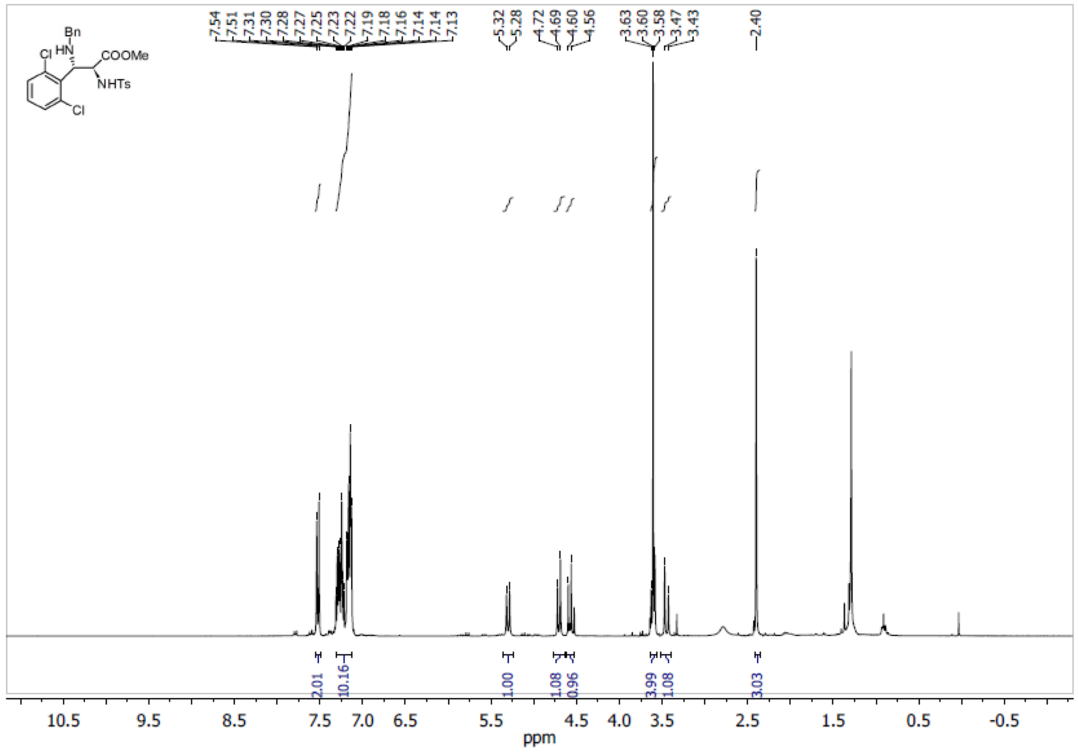

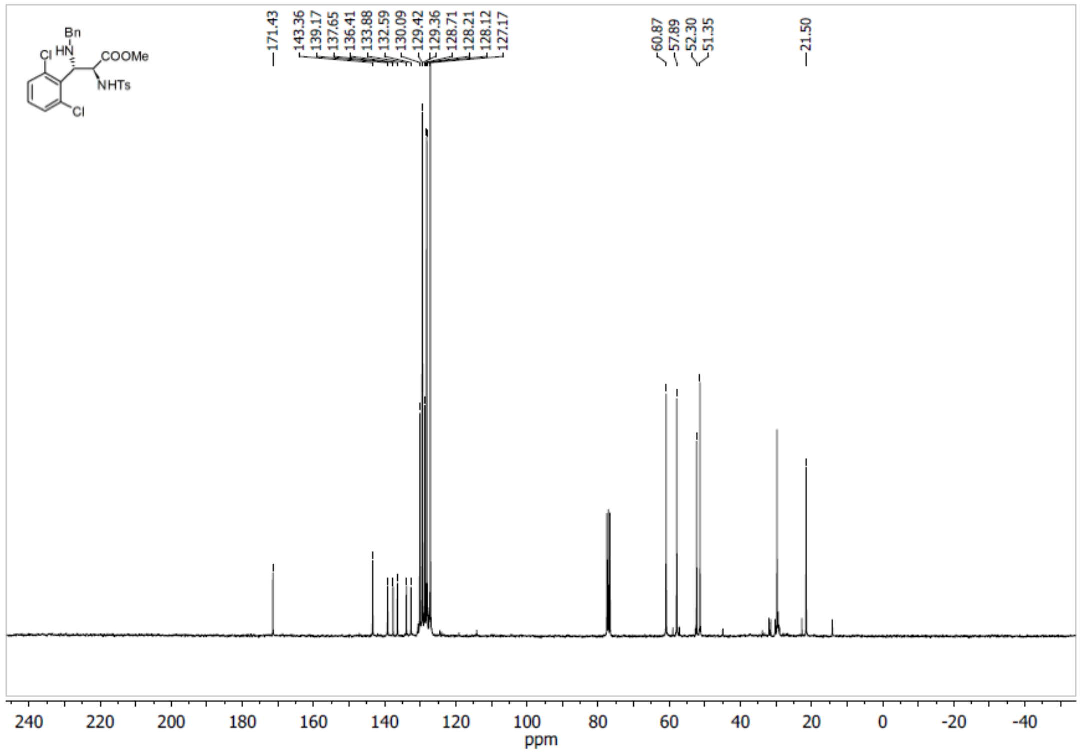

^{1}H and ^{13}C NMR of **5n** ($CDCl_3$)

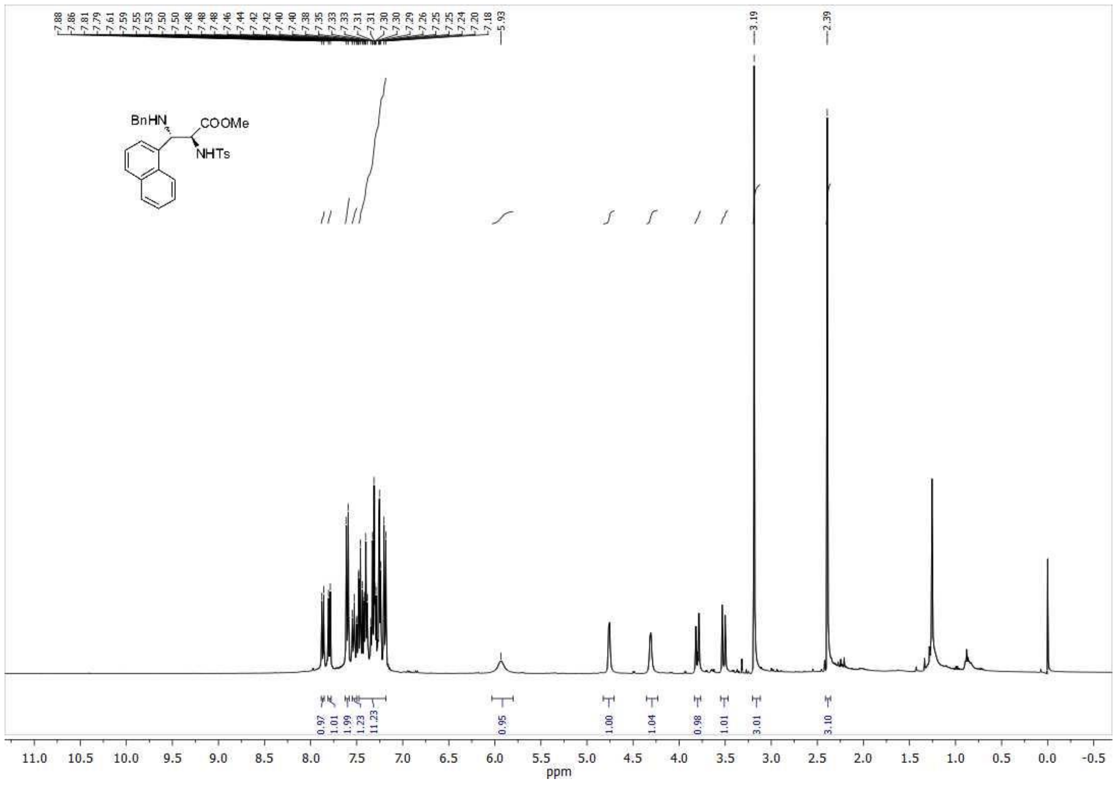

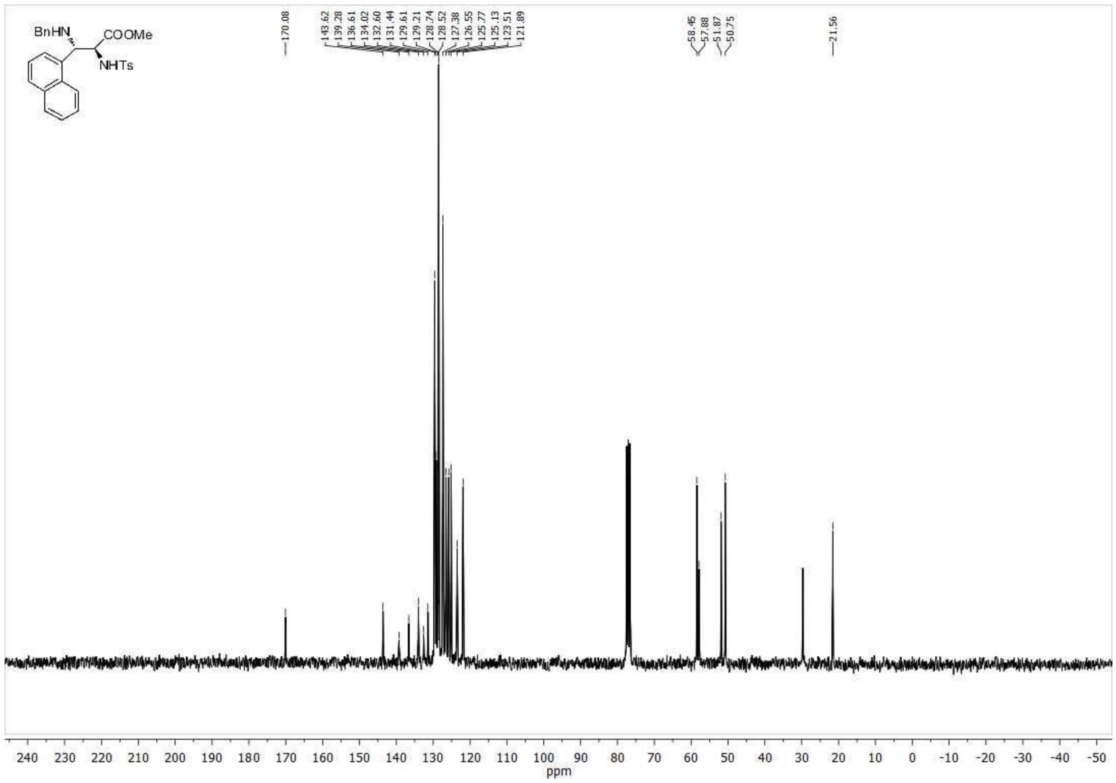

^{1}H and ^{13}C NMR of **5o** ($CDCl_3$)

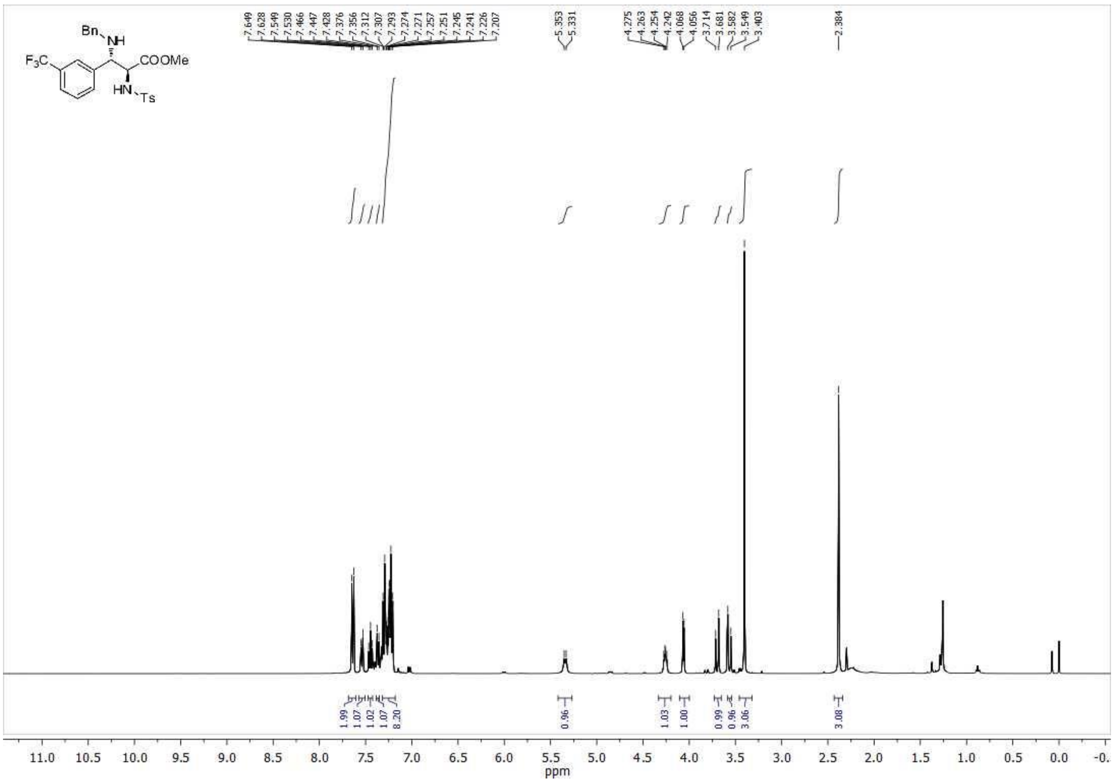

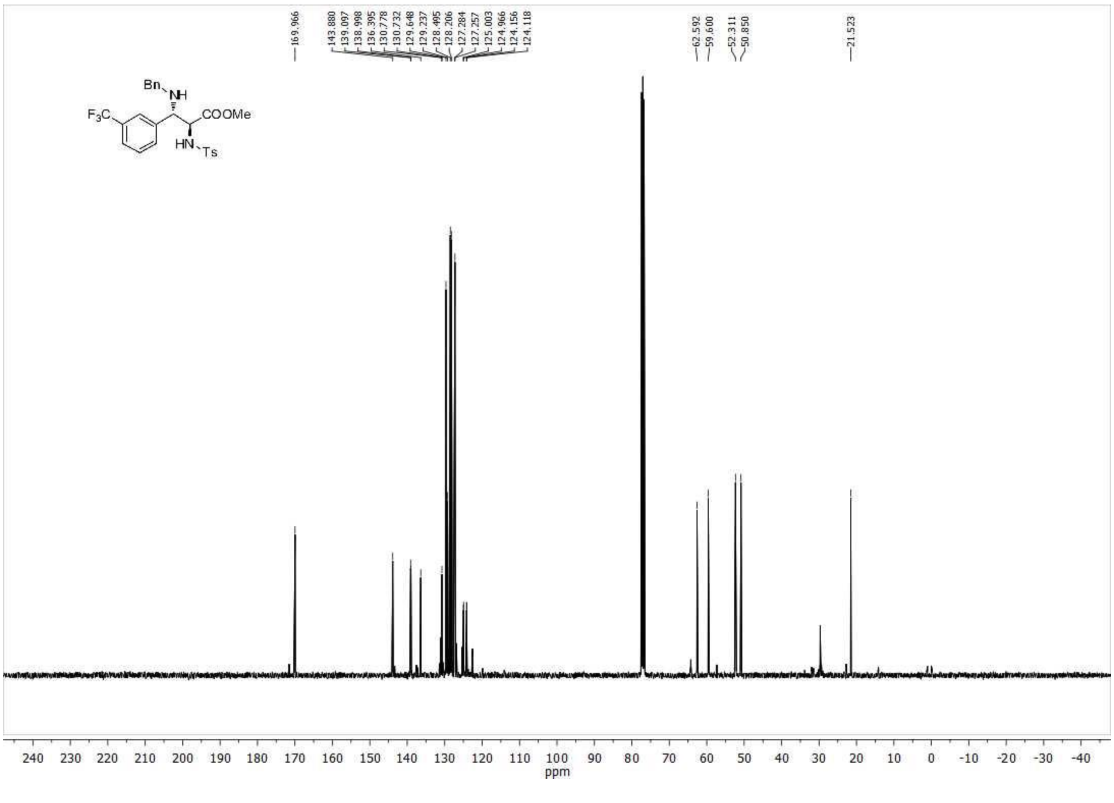

^{1}H and ^{13}C NMR of **5p** ($CDCl_3$)

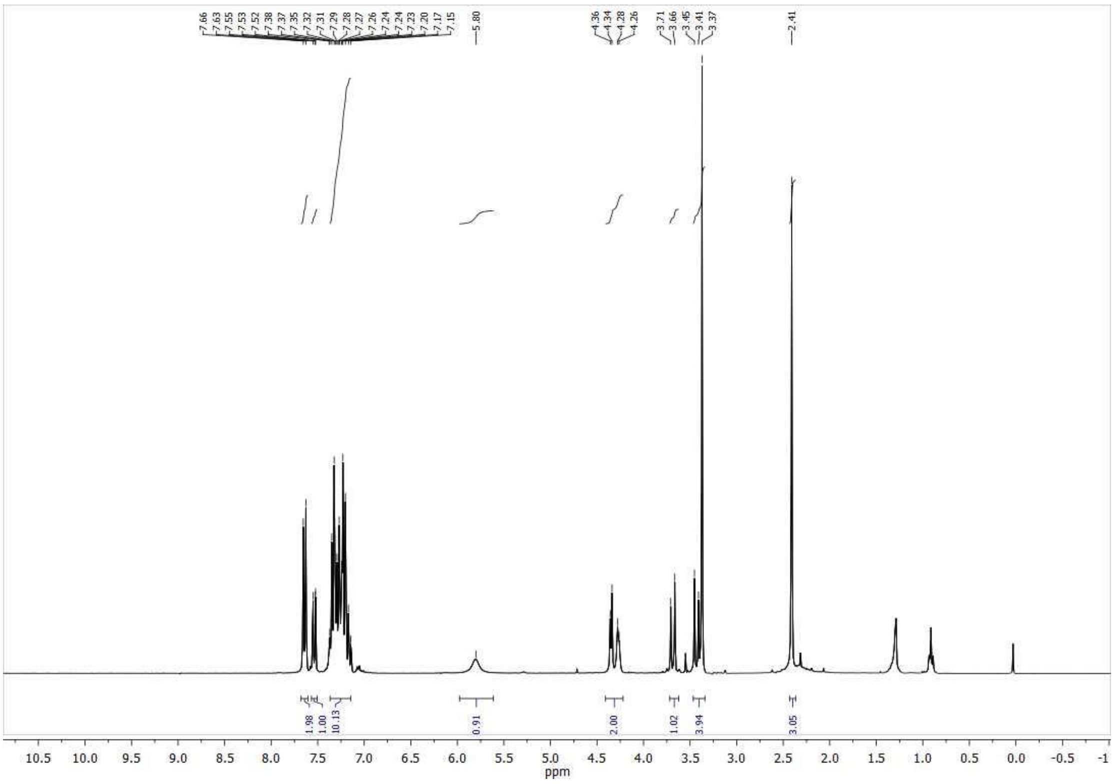

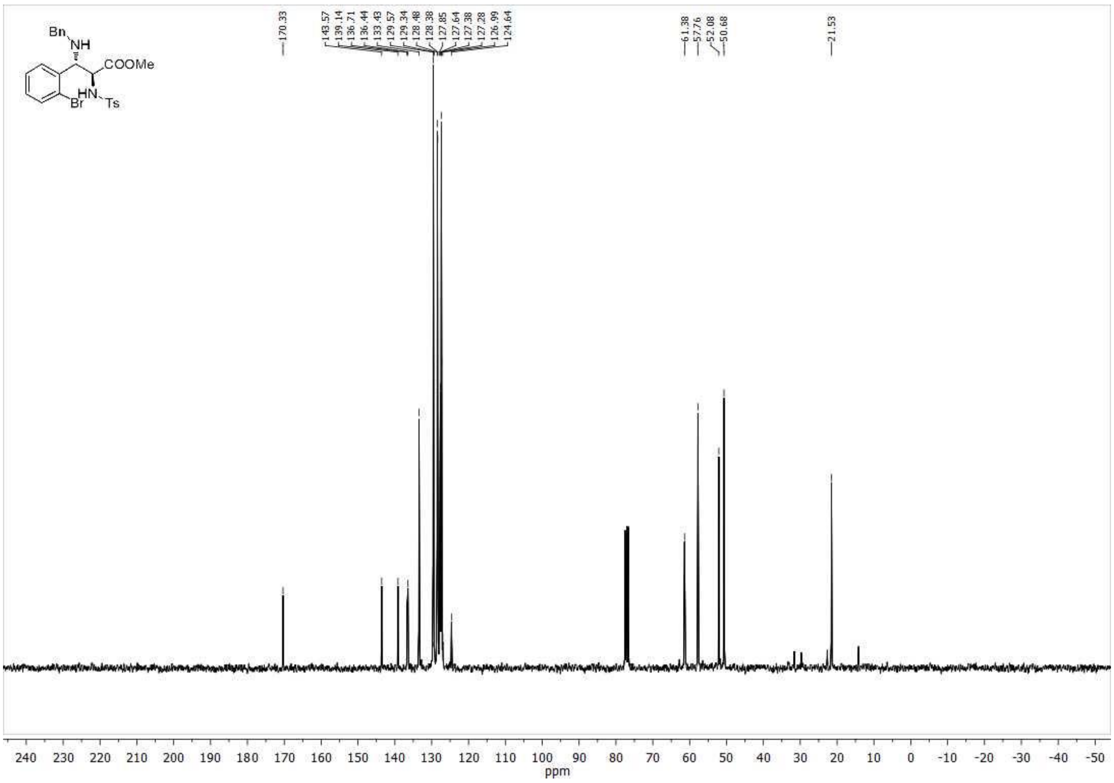

Supplement: File 1 — Experimental details and spectral data. [file Beilstein_J_Org_Chem-10-1802-s001.pdf]
